# Mapping the follicle-specific regulation of extracellular vesicle-mediated microRNA transport in the southern white rhinoceros (*Ceratotherium simum simum*)^†^

**DOI:** 10.1093/biolre/ioae081

**Published:** 2024-05-22

**Authors:** Ahmed Gad, Nico G Menjivar, Rachel Felton, Barbara Durrant, Dawit Tesfaye, Elena Ruggeri

**Affiliations:** Animal Reproduction and Biotechnology Laboratory (ARBL), Department of Biomedical Sciences, College of Veterinary Medicine and Biomedical Sciences, Colorado State University, Fort Collins, CO 80523, USA; Department of Animal Production, Faculty of Agriculture, Cairo University, Giza 12613, Egypt; Animal Reproduction and Biotechnology Laboratory (ARBL), Department of Biomedical Sciences, College of Veterinary Medicine and Biomedical Sciences, Colorado State University, Fort Collins, CO 80523, USA; Reproductive Sciences, Conservation Science Wildlife Health, San Diego Zoo Wildlife Alliance, Escondido, CA 92027, USA; Reproductive Sciences, Conservation Science Wildlife Health, San Diego Zoo Wildlife Alliance, Escondido, CA 92027, USA; Animal Reproduction and Biotechnology Laboratory (ARBL), Department of Biomedical Sciences, College of Veterinary Medicine and Biomedical Sciences, Colorado State University, Fort Collins, CO 80523, USA; Reproductive Sciences, Conservation Science Wildlife Health, San Diego Zoo Wildlife Alliance, Escondido, CA 92027, USA

**Keywords:** rhinoceros, ovarian follicle, follicular fluid, extracellular vesicles, miRNA

## Abstract

Efforts to implement effective assisted reproductive technologies (ARTs) for the conservation of the northern white rhinoceros (NWR; *Ceratotherium simum cottoni*) to prevent its forthcoming extinction, could be supported by research conducted on the closely related southern white rhinoceros (SWR; *Ceratotherium simum simum*). Within the follicle, extracellular vesicles (EVs) play a fundamental role in the bidirectional communication facilitating the crucial transport of regulatory molecules such as microRNAs (miRNAs) that control follicular growth and oocyte development. This study aimed to elucidate the dynamics of EV-miRNAs in stage-dependent follicular fluid (FF) during SWR ovarian antral follicle development. Three distinct follicular stages were identified based on diameter: Growing (G; 11–17 mm), Dominant (D; 18–29 mm), and Pre-ovulatory (P; 30–34 mm). Isolated EVs from the aspirated FF of segmented follicle stages were used to identify EV-miRNAs previously known via subsequent annotation to all equine (*Equus caballus*; eca), bovine *(Bos taurus*; bta), and human (*Homo sapiens*; hsa) miRNAs. A total of 417 miRNAs were detected, with 231 being mutually expressed across all three stages, including eca-miR-148a and bta-miR-451 as the top highly expressed miRNAs. Distinct expression dynamics in miRNA abundance were observed across the three follicular stages, including 31 differentially expressed miRNAs that target various pathways related to follicular growth and development, with 13 miRNAs commonly appearing amidst two different comparisons. In conclusion, this pioneering study provides a comprehensive understanding of the stage-specific expression dynamics of FF EV-miRNAs in the SWR. These findings provide insights that may lead to novel approaches in enhancing ARTs to catalyze rhinoceros conservation efforts.

## Teaser

Stage-specific miRNA cargo encapsulated within follicular fluid-derived extracellular vesicles possess intrinsic functions to modulate cell–cell communication during follicular growth in the rhinoceros.

## Graphical Abstract

**Graphical Abstract:**
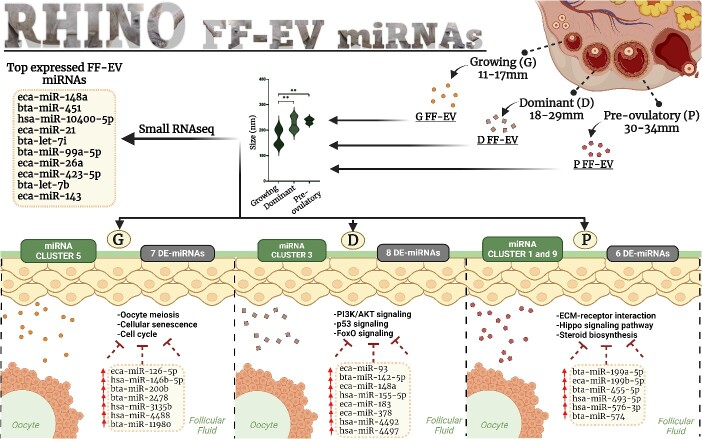

## Introduction

Wild rhinoceros populations are facing extinction due to constant threats; increased conservation measures are critically necessary to protect these species. As one of the few mega vertebrate herbivores living in the world, rhinoceros play a vital role as a keystone species of the African landscape. With just two non-reproductive living individuals, global attention has been focused on rescuing the northern white rhinoceros (NWR) from extinction [1, 2]. Conservation efforts have been focused on using the NWR’s closest relative, the SWR to overcome rhinoceros population decline and to develop effective propagation strategies using assisted reproductive technologies (ARTs). Numerous ART procedures have been developed for different rhinoceros species, including ovum pickup (OPU), which enables oocytes to be retrieved for *in vitro* maturation and intracytoplasmic sperm injection, resulting in blastocyst production and cryopreservation for future use [2]. In addition, advanced ARTs, such as stem cell-associated techniques and artificial gamete production, could also pose promising strategies to support conservation efforts in rhinoceros [3].

Unfortunately, working with the rhinoceros comes with inherent management challenges and limited procedure opportunities. Therefore, to advance our reproductive knowledge and develop conservation technologies, domestic species are used to aid in the lesser accessible species, including the NWR and SWR. The domestic horse is phylogenetically related to the white rhinoceros, hence it can be a valuable model for reproductive studies [2]. Other domestic species, as well as humans, may also provide scientific context to advance rhinoceros reproductive knowledge and promote conservation efforts, although there is less comparative and translational work reported. Like the domestic horse, the white rhinoceros is a mono-ovulatory species, exhibiting a 3- to 5-day period of estrus behavior [4, 5], accompanied by the growth and ovulation of a single follicle (30–40 mm) [6], preceeded by a single pre-ovulatory LH surge. Throughout folliculogenesis, many antral follicles exist, segmented into multiple stages based on follicle diameter size (also known as selection, dominance, and ovulation). The nature of follicle dynamics and subsequent mechanisms of development are largely based on a series of systemic processes involving hormonal interaction and various intrafollicular constituents [7].

In the rhinoceros, serial ultrasound examinations have provided detailed characterizations of normal follicular growth and ovulation, as well as the documentation of anovulatory cycles amidst other irregularities. In captivity, cyclic females exhibit estrous cycles from 20 (similar to the horse) to 80 days in duration [8]. These two cycle lengths are deemed short (clustered around 30 days) and long (clustered around 70 days), but no mechanistic difference has been found to explain these differences in cycle length [4, 6, 9–13]. It was previously thought that the long cycle was either infertile or subfertile, but recent work has resulted in established pregnancies from long estrous cycles [8]. Regardless of the cycle length, the SWR cycle has been successfully manipulated with gonadotropin-releasing hormone (GnRH) to induce ovulation when follicles reach 30–35 mm [6, 8, 14, 15]. Moreover, in domesticated species like horses and cattle, GnRH treatment is correspondingly used to obtain high-quality oocytes and achieve greater pregnancy rates [16–18]. Ultimately, this unusual pattern of an irregular estrous cycle length, potentially complicates the development of ARTs in the SWR.

More recently, in a variety of domesticated species, including the horse, primary attention has focused on the role of EVs in governing intrafollicular communication, follicle dynamics, and reproductive function. Extracellular vesicles are lipid-bound nanoparticles secreted by cells [19] and encompass a heterogenous population of exosomes, microvesicles, and apoptotic bodies, which are primarily differentiated by their size and mode of biogenesis. These vesicles contain lipids, nucleic acids, and proteins, and are a functional system regulating the bidirectional communication between cells present within most body fluids. In addition, EVs are evolutionarily conserved structures secreted from a variety of cell types through exocytosis [20]. Previously discovered within the FF of various mammalian species and presiding over events including folliculogenesis and oogenesis [21–23], EVs of follicular, oviductal, and uterine fluid origin [24–28] have suitably proved to be beneficial in promoting oocyte competence and embryo development [29–31].

Recently, stage-specific expression profiles of follicular fluid EV-miRNAs during equine follicular development were performed using high-throughput miRNA sequencing to elucidate the patterns of EV-miRNAs and their association during specific stages of follicle development [24]. As such, in this study, critical miRNAs regulating follicular development were identified, proposing novel approaches using EV-mediated signaling to improve *in vitro* technological outcomes that span a multitude of species. These findings could be used for comparative approaches in understanding the potential role of EV-coupled miRNA dynamics in regulating follicular growth in the rhinoceros. Furthermore, the EV-miRNA patterns observed during follicle development could duly be used to distinguish physiological and pathological reproductive conditions, aid in defining fertility parameters, and to identify targets of future intervention, ultimately improving ART outcomes.

## Materials and methods

### Animal management and transrectal ovum pick up

Animals for this study were housed and managed at the San Diego Zoo Wildlife Alliance’s Safari Park in Escondido, CA. All experimental procedures and methods involving the specific use of animals were reviewed and approved by the San Diego Zoo Wildlife Alliance’s Institutional Animal Care and Use Committee (IACUC; protocol number #18-018, United States Department of Agriculture Certificate #93-R-0151).

For this study, two southern white rhinoceros females were stimulated prior to transrectal OPU similar to previous protocols [32, 33], facilitating the *in vivo* collections of FF aspirate from antral follicles of distinct sizes. Females orally received synthetic chlormadinone acetate (CMA) at 3 mg/day for 35 days. Two days after CMA withdrawal, both animals received 3 mg of Deslorelin (GnRH analog) followed by 2 mg of Deslorelin every 48 h prior to OPU (7 mg total) via injection. Both rhinoceroses were anesthetized using a combination of etorphine (2 mcg/kg), butorphanol (20–24 mcg/kg), medetomidine (23–25 mcg/kg), and azaperone (14–16 mcg/kg) administered intramuscularly using a remote drug delivery system. Propofol was administered intravenously during the initial positioning to facilitate intubation (0.5 mg/kg). Concluding collection procedures, anesthesia reversal was achieved using atipamezole delivered intramuscularly at a 5:1 ratio to medetomidine (116–125 mcg/kg) and naltrexone delivered intramuscularly at a target 50:1 ratio to etorphine (53–61 mcg/kg).

**Figure 1:**
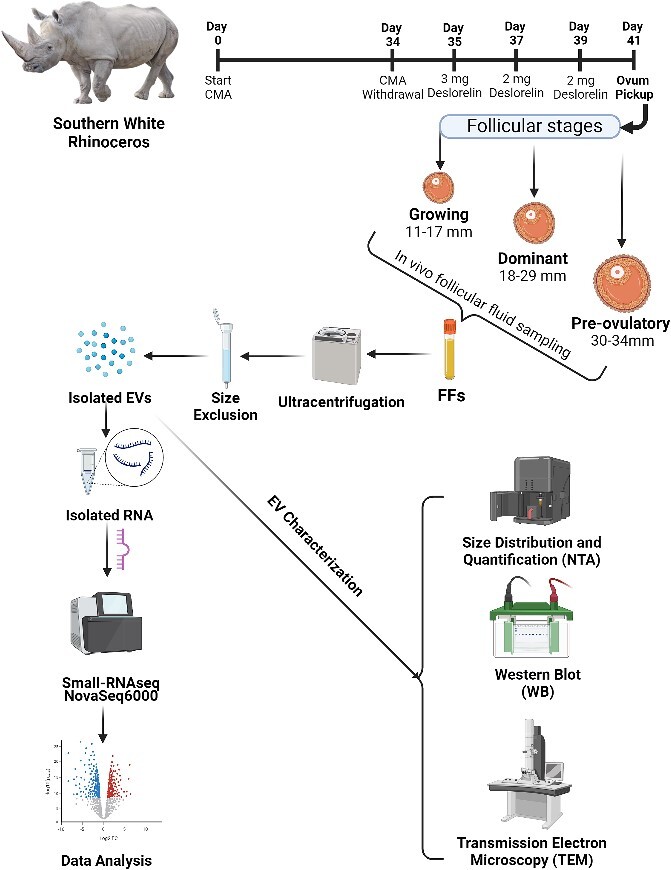
Schematic experimental illustration of the data analysis pipeline. Experimental pipeline outlining the design and workflow for the analysis of extracellular vesicle-coupled miRNAs from stage-specific rhinoceros FF. Given the rairity of the sample collections, following *in vivo* FF collections and subsequent EV isolation, the first-factor variable of differences in individual animals were removed. Total RNA including small RNAs were isolated from equal volumes of pooled EV technical replicates of two biologically dissimilar females (three pooled replicates/follicle stage, pool consisting of two animals).

Following fecal removal, rinsing, and disinfection, OPU was achieved using a customized, ultrasound-guided probe enclosing three double-lumen needles. Follicle contents were aspirated, and each follicle was rinsed with a warmed (37°C) flushing solution (Vigro) containing 12.5 I.U./mL of heparin. The complete experimental design is illustrated in Figure 1.

### Ovarian follicle grouping and FF collection

Prior to ultrasound-guided aspirations, antral follicles were assigned to one of three predetermined categories based on their diameters: growing (G; 11–17 mm), dominant (D; 18–29 mm), and pre-ovulatory (P; 30–34 mm), adapted from previous work [24]. Follicle aspirates were maintained at 37°C and immediately transported to the laboratory, where they were filtered through a 0.22-μm embryo filter (Professional Embryo Transfer Supply Inc.). Prior to storage, EV-bearing FF was centrifuged at 300 × g for 10 min, with the uppermost aqueous phase transferred and re-centrifuged at 2000 × g for 10 min, and 16 500 × g for 30 min. The final supernatant was passed through a 0.20-μm sterile syringe filter, removing particles larger than 200 nm in diameter, and stored at −80°C for further processing [34].

### EV isolation and characterization

EVs were isolated using the dual-targeted approach of ultracentrifugation followed by precipitation and elution through size exclusion chromatography columns. Initially, in three technical replicates, frozen-thawed aliquots of 2 mL of FF aspirate from individual animals were subjected to ultracentrifugation using an SW 55Ti rotor on the Optima XE-90 Ultracentrifuge (Beckman Coulter; Pasadena, CA, USA) system at 120 000 × g for 70 min at 4°C, as previously described [28, 31]. Pelletized EVs were then washed with 3 mL of Dulbecco’s Phosphate Buffered Saline without calcium and magnesium chloride [DPBS (1X)] (Sigma-Aldrich; St. Louis, MO, USA) and ultracentrifuged once more under the same parameters. Following ultracentrifugation, washed EV pellets were resuspended in Exo-spin™ Buffer and left to precipitate overnight at 4°C prior to processing through Exo-spin™ mini columns (CELL guidance systems; St. Louis, MO, USA) according to the manufacturer’s protocol. Final 180-μL EV aliquots were then stored at −80°C for RNA extraction.

Identification and characterization methods of EVs (western blotting, transmission electron microscopy, and nanoparticle tracking analysis) were carried out as recommended by the International Society for Extracellular Vesicles in the proposed Minimal Information for Studies of Extracellular Vesicles (“MISEV”) guidelines [35, 36]. Detailed experimental procedures have been submitted to the EV-TRACK knowledgebase (https://evtrack.org/) (EVTRACK ID: EV240017) [37].

#### Western blot

Isolated EV samples suspended in DPBS (1X) were combined with RIPA Ready to use [RIPA-RTU; 890-μL RIPA Buffer (Sigma R0278), 10-μL phosphatase inhibitor cocktail (Fisher 78420), 100-μL10X protease inhibitor cocktail (Sigma 11836170001)] to detect EV and cellular marker proteins. Protein lysate mixtures were vortexed for 30 s and kept on ice prior to suspension in 4x Laemmli Sample Buffer (Bio-Rad 1610747) and 2-Mercaptoethanol (Bio-Rad 1610710). Loading mixtures were then boiled at 95°C for 5 min, and protein samples were separated using Sodium Dodecyl Sulfate-PolyAcrylamide Gel Electrophoresis on a gradient (4–15%) Mini-PROTEAN TGX Stain-Free Precast Gels (Bio-Rad 4568084) and transferred to a 0.20-μm pore nitrocellulose membrane (Bio-Rad 1620112). Membranes were blocked for 1 h at room temperature in 5% (w/v) Non-Fat Dry Milk (Bio-Rad 1706404) in 1X Tris-buffered saline-Tween 20 (TBS-T) buffer and further incubated overnight at 4°C with respective primary antibodies. Membranes were then incubated with the suitable secondary antibody conjugated with horseradish peroxidase (System Biosciences; Goat anti-Rabbit HRP, 1:200 dilution) for 1 h at room temperature. Membranes were each probed and developed once with SuperSignal^™^ West Pico PLUS Chemiluminescent Substrate (ThermoFisher Scientific 34,579) and imaged using the ChemiDoc XRS+ chemiluminescence system with Image Lab^™^ Software (version 6.0.1 build 34) (Bio-Rad).

The following rabbit anti-human antibodies were used for western blot: rabbit polyclonal TSG101 (System Biosciences; EXOAB-TSG101-1; 1:100 dilution), rabbit polyclonal HSP70 (System Biosciences; EXOAB-Hsp70A-1; 1:100 dilution), and rabbit polyclonal Cytochrome C (Sino Biological; 102139-T42; 1:200 dilution). All antibodies were diluted in 5% (w/v) Non-Fat Dry Milk (Bio-Rad 1706404) in TBS-T.

#### Transmission electron microscopy

The morphological shape and size of EVs were determined using a particle beam of electrons for visualization on a FEI/TFS Tecnai T12 Spirit transmission electron microscope (FEI Company; Hillsboro, OR, USA), operating at 100 kV, with an AMT CCD. Briefly, a small volume of isolated EV samples (6-8 μL) was applied to a carbon-coated, copper mesh grid for 1–2 min., followed by negative staining with 2% aqueous uranyl acetate. After fixing, samples were allowed to dry prior to imaging. Multiple frames were visualized per sample with approximately 15–20 captured for assessment.

#### Nanoparticle tracking analysis

The size distribution and collective particle concentration in isolated EV samples were analyzed using a ZetaView® QUATT 4 Nanosight Instrument (Particle Metrix). Briefly, isolated EVs were diluted in sterile DPBS (1X) and injected into the device for analysis. Measurements were taken at 11 positions via scatter mode using a 488-nm laser. Analysis of EV concentrations was calculated using the Zetaview Software (version 8.05.12 SP1) and re-analyzed based on the dilution factors to reveal the approximate number of particles/mL for each sample.

### Exosomal RNA extraction, library preparation, and sequencing

Total RNA to include miRNAs were isolated from equal volumes of pooled EV technical replicates of two biologically dissimilar females (three pooled replicates/follicle stage, pool from 2 animals) using the Norgen Exosomal RNA Isolation kit (Norgen Biotek Corp.; Thorold, ON, CA.) following the instructions for the Mini Kit provided by the manufacturer, and as carried out previously [24, 38]. Objective measurements for RNA quality using RINs (RNA Integrity Number), concentration, and size distribution were characterized using an Agilent RNA 6000 Pico kit in an Agilent 2100 Bioanalyzer (Agilent Technologies; Santa Clara, CA, USA). Small-RNA library preparations for Next Generation Sequencing were prepared by Novogene Co., LTD (Sacramento, CA, USA) using a TruSeq Small RNA Library Prep Kit (Illumina RS-200) according to the manufacturer’s instructions. Library quantity and quality assessments were performed using a Qubit® DNA HS Assay Kit on a Qubit® 2.0 Fluorometer and an Agilent DNA High Sensitivity Kit in an Agilent 2100 Bioanalyzer, respectively. Explicit library concentrations were calculated using quantitative PCR measures then pooled in equimolar ratios and sequenced in a NovaSeq6000 sequencing instrument (Illumina) as single-end (50 bases) reads.

### Bioinformatic analysis and identification of miRNA clustering

FASTQ files were generated for individual samples using the software bcl2fastq (Illumina) with quality assurance performed using FastQC tool version 0.11.9. Raw FASTQ files and processed comma-separated value (CSV) files have been deposited into NCBI’s Gene Expression Omnibus (GEO) and are accessible through the GEO Series accession number (GSE246260). Formal analysis of the data was conducted using the CLC Genomics Workbench software, version 21 (www.qiagenbioinformatics.com). After removing adapter sequences, raw sequencing reads were trimmed based on quality score (Q-score > 30), ambiguous nucleotides (maximum two nucleotides allowed), and read length (≥15 nucleotides). As miRNAs have not been sequenced for the rhinoceros species, reads were annotated against the equine, bovine, and human precursor and mature miRNAs listed in the mirBase database (release 22) using the CLC Genomics Workbench Quantify miRNA tool after applying default software parameters and allowing a maximum of two mismatches within the read. We used these three species to annotate the rhinoceros’s sequence reads as they have well-annotated genomes and miRNAs and well-developed ARTs, with equine given priority based on the phylogenetic relationship [39]. Normalization of raw expression data was conducted using the trimmed mean of the M-values method (TMM normalization) [40] and correspondingly presented as TMM-adjusted Counts Per Million (CPM). Any miRNA with a CPM value greater than 10 in a minimum of two out of the three biological replicates of each group was considered as expressed. Additionally, the expression analysis comparison was performed using the CLC Genomics Workbench Differential Expression tool. Differentially expressed (DE) miRNAs were determined on the criteria of exhibiting a fold change (FC) ≥ 2 and p-adjusted value (FDR) < 0.1 [41]. miRNAs were grouped in different cluster patterns according to their expression profiles among the different developmental stages using the Mfuzz Bioconductor package [42].

### miRNA-target gene prediction and ontological classification

Target genes of the DE-miRNAs were identified using the human DE-miRNAs and the human homologous miRNAs for the equine and bovine DE-miRNAs to cover all possible validated and predicted target genes appearing in the miRWalk database [43]. Validated target genes from miRTarBase (version 7.0) and predicted commonly targeted genes by TargetScan (version 7.1) and miRDB (release 5.0) were selected for ontological classification analysis using the DAVID bioinformatics web tool (https://david.abcc.ncifcrf.gov/). Significant pathways and biological processes were determined from the KEGG pathway database [44], and the GOTERM_BP_DIRECT annotation set, respectively. The interaction network of the DE-miRNAs from the different comparisons were constructed using Cytoscape [45].

## Results

### Rhinoceros EVs bear conventional EV-like physical and biochemical traits

**Figure 2:**
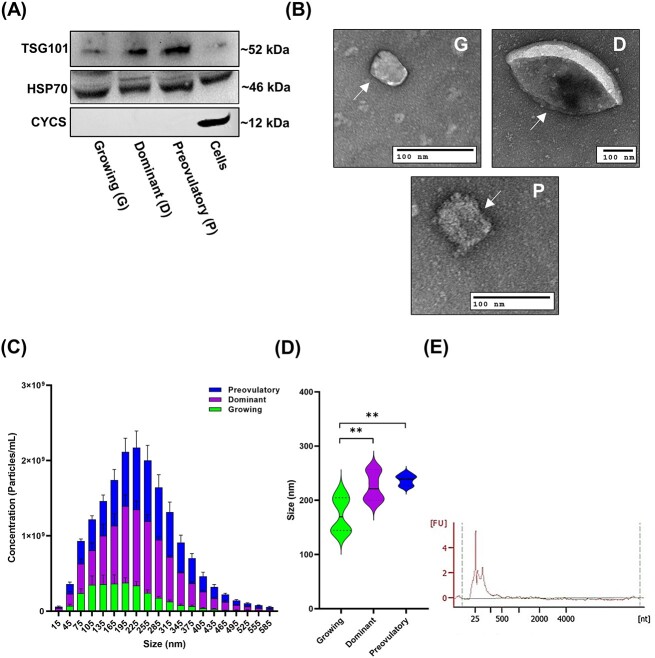
Rhinoceros extracellular vesicle morphological and biochemical validation. (A) Immunoblots indicating traditionally expressed EV-associated marker proteins (TSG101 and HSP70), and the absence of cellular contamination marker (CYCS). (B) Representative transmission electron microscopy images revealing the ultrastructure cup-like morphology of rhinoceros FF-derived EVs, indicated by arrows. Scale bars: 100 nm. Nanoparticle tracking analysis of the concentration (C) and size distribution (D) of rhinoceros FF-derived EVs from diverging stages. (E) Representative graphical overview of the quality and integrity of exosomal RNA samples used for small RNA sequencing.

Stage-specific FF was collected from two female rhinoceros’ and subjected to a dual-targeted approach to isolate high-grade EVs for small RNA sequencing, as outlined in the workflow diagram (Figure 1). The analysis of the isolated EVs and independent rhinoceros granulosa cells revealed the presence of EV markers (TSG101 and HSP70) in all samples, assessed by western blot analysis, as well as the absence of cellular mitochondrial protein marker cytochrome c (CYCS) from the EV samples (Figure 2A). Transmission electron microscopy images captured of pooled EV samples from each respective stage, revealed that rhinoceros EVs exhibit hallmark cup-like morphology, characteristics indicative of long-established EVs (Figure 2B). Additionally, we evaluated six technical replicates of EVs [three per follicle stage, per individual animal (*n* = 2)], and according to nanoparticle tracking analysis, EVs collected from stage-specific FF differ in concentration (Avg. Conc.: G = 2.93E ± 09, D = 8.17E ± 09, P = 7.05E ± 09) and differ in size [G = 173.78 nm, D = 225.76 nm (*p* = 0.0072), P = 237.41 nm (*p* = 0.0015)], compared to those EVs collected from growing follicles (Figure 2C and D). The quality and integrity of the isolated exosomal RNA samples were used to sequence small RNAs (n = 9) and were additionally analyzed with a representative electropherogram revealing peaks for small RNAs amid EVs with the absence of 18S and 28S cellular ribosomal RNAs (Figure 2E).

### Expression profiles and DE miRNAs

**Figure 3:**
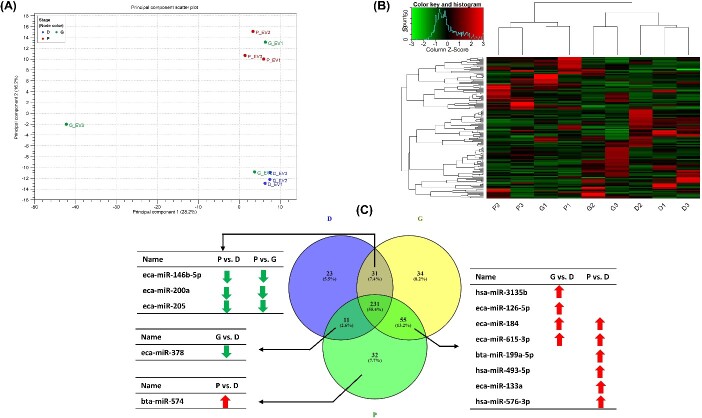
Small-RNA sequencing data overview. (A) Principal component analysis. (B) Heatmap and hierarchical clustering of expressed miRNAs. (C) Venn diagram for commonly and exclusively expressed miRNAs in the FF EVs at different stages of follicular development. MicroRNAs with a value of CPM >10 in at least two replicates of the three biological replicates was considered detected. Exclusively expressed miRNAs that exhibited a significant difference are presented in the Venn diagram with arrows indicating significantly up or downregulated miRNAs in the corresponding comparison. G: Growing; D: dominant; P: preovulatory.

A total of nine small-RNA libraries were constructed with an average of 18 million reads per library that passed the QC parameters. An average of 0.4% of reads were annotated to the equine, bovine, and human miRNAs from the mirBase database (Supplementary Table S1). Principal component analysis (PCA) and hierarchical heatmap exhibited clear clusterings of the D and P replicates, while this was not the case for the replicates of the G group (Figure 3A and B). A total of 351, 296, and 329 miRNAs were expressed (>10 CPM in at least two replicates) in the G, D, and P groups, respectively, with 231 miRNAs being commonly expressed across the three groups (Figure 3C). The top 20 highly expressed miRNAs are presented in Table 1 and the complete list of all expressed miRNAs is presented in Supplementary Table S2. Among the top 20 expressed miRNAs, 13 miRNAs were commonly detected in the three groups. Interestingly, eca-miR-148a and bta-miR-451 were among the top three miRNAs in the three groups (Table 1). Among the exclusively expressed miRNAs, or the ones that were expressed in only two groups, 13 miRNAs exhibited differences in either one or two comparisons, as shown in Figure 3(C).

**Table 1:** List of the top 20 most abundant miRNAs amidst EVs obtained from rhinoceros FFs at different follicular stages

**miRNA**	**G CPM**	**miRNA**	**D CPM**	**miRNA**	**P CPM**
**bta-miR-11980**	185 839.3	**eca-miR-148a**	872 829.2	**eca-miR-148a**	94 882.4
**eca-miR-148a**	85 922.45	**bta-miR-451**	96 984.29	**hsa-miR-10400-5p**	54 664.73
**bta-miR-451**	83 159.52	**hsa-miR-10400-5p**	91 187.32	**bta-miR-451**	38 476.65
**bta-miR-2478**	50 026.85	**eca-miR-21**	45 580.34	**bta-miR-99a-5p**	38 245.91
**hsa-miR-3168**	47 238.03	**eca-miR-122**	32 587.91	**eca-miR-99a**	30 796.38
**eca-miR-21**	34 231.73	**eca-miR-486-5p**	30 169.45	**bta-let-7i**	24 071.29
**bta-let-7i**	29 962.76	**bta-let-7i**	28 037.44	**eca-miR-21**	23 430.8
**hsa-miR-10400-5p**	24 243.74	**hsa-miR-3168**	26 703.2	**eca-miR-10b**	21 032.38
**bta-miR-99a-5p**	24 046.17	**bta-miR-99a-5p**	23 336.02	**eca-miR-143**	20 767.12
**eca-miR-26a**	20 774.92	**eca-miR-26a**	21 300.4	**eca-miR-26a**	18 064.75
**eca-miR-1**	20 262.69	**bta-miR-192**	17 449.7	**bta-let-7b**	17 731.89
**eca-let-7g**	16 584.2	**eca-let-7g**	14 136.19	**eca-let-7a**	16 031.34
**eca-let-7f**	16 230.58	**eca-miR-423-5p**	13 883.41	**bta-miR-192**	15 927.29
**eca-miR-486-5p**	16 206.56	**bta-let-7b**	13 142.76	**eca-miR-122**	15 727.23
**bta-miR-151-3p**	15 349.26	**eca-miR-126-3p**	12 884.64	**eca-miR-100**	15 330.03
**eca-miR-143**	13 270.89	**eca-miR-143**	11 875.72	**eca-miR-1**	13 948.54
**eca-miR-423-5p**	12 834.61	**eca-let-7f**	11 162.91	**eca-miR-128**	12 321.1
**bta-let-7b**	12 829.24	**eca-miR-128**	10 955.84	**eca-let-7f**	12 199.41
**eca-miR-122**	12 587.2	**bta-miR-151-3p**	10 827	**bta-miR-151-3p**	11 359.67
**eca-miR-100**	11 683.97	**eca-miR-10b**	10 642.12	**eca-miR-423-5p**	9912.52

miRNA: microRNA; CPM: counts per million; G: growing; D: dominant; P: pre-ovulatory.

**Figure 4:**
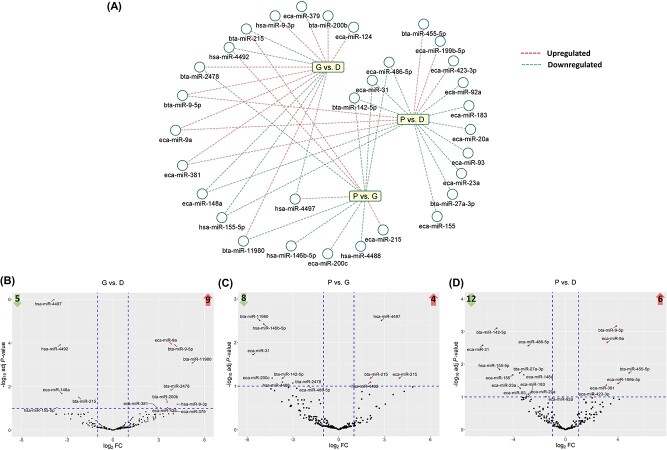
DE miRNAs. (A) All DE-miRNAs from the three different comparisons. Up- and down regulated miRNAs from each comparison are presented as red and green lines, respectively. Volcano plots of expressed miRNAs. Up- (upper right), and downregulated (upper left) miRNAs in (B) G vs. D, (C) P vs. G, and (D) P vs. D comparisons. The number of up and downregulated miRNAs in each comparison are presented on the up and down arrows, respectively. G: growing; D: dominant; P: preovulatory.

In addition, a total of 31 miRNAs, among the 231 commonly expressed miRNAs, were DE (FC ≥ 2, FDR < 0.1) in the three different comparisons (Figure 4A). The DE-miRNAs in the G vs. D, P vs. G, and P vs. D comparisons are presented in Figure 4(B), (C), and (D), respectively and in the Supplementary Table S3. Out of the 31 DE-miRNAs, 13 commonly appeared in atleast two different comparisons. Three miRNAs, eca-miR-486-5p, eca-miR-31, and bta-miR-142-5p were downregulated in the P group compared to both the G and D groups. In the G group, bta-miR-215, hsa-miR-4492, and hsa-miR-4497 were downregulated, while bta-miR-2478 and bta-miR-11980 were upregulated compared to both the P and D groups. In the D group, bta-miR-9-5p, eca-miR-9a, and eca-miR-381 were downregulated, while eca-miR-148a and hsa-miR-155-5p were upregulated compared to both the G and P groups (Figure 4).

### miRNA cluster analysis

**Figure 5:**
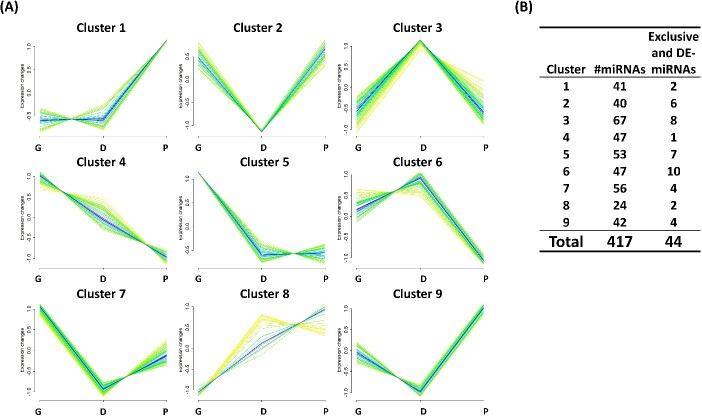
Clustering analysis of EV-coupled miRNAs across the stages of follicular development. (A) Nine different miRNA expression patterns in the FF EVs during follicular development. (B) The counts of total and DE miRNAs in each cluster. G: growing; D: dominant; P: preovulatory.

The abundance of EV-coupled miRNAs across the stages of follicular development was sorted according to the abundance in each stage of development and shown to have distinct clustering patterns. Cluster analysis showed that the miRNAs expressed in the FF-EVs were clustered into nine different patterns (Figure 5A, Supplementary Table S4). Each cluster pattern was composed of a variable number of miRNAs, as well as, DE-miRNAs, in which, clusters 3, 5, and 6 contained the highest numbers of DE-miRNAs compared to the other clusters (Figure 5B).

**Figure 6:**
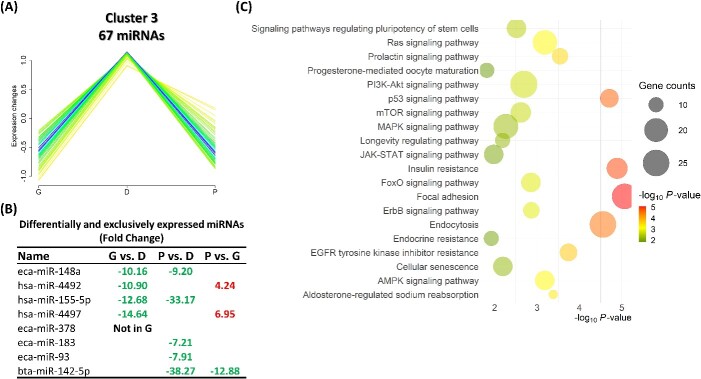
Cluster 3 consists of 67 miRNAs. (A) A group of 67 miRNAs exhibited an increase in their expression from the G until the D stage and then a reduction at the P stage. (B) Among this cluster, eight miRNAs were differentially or exclusively expressed amidst the different comparisons. (C) Pathways associated with the exclusive and DE-miRNA target genes of this cluster. G: growing; D: dominant; P: preovulatory.

**Figure 7:**
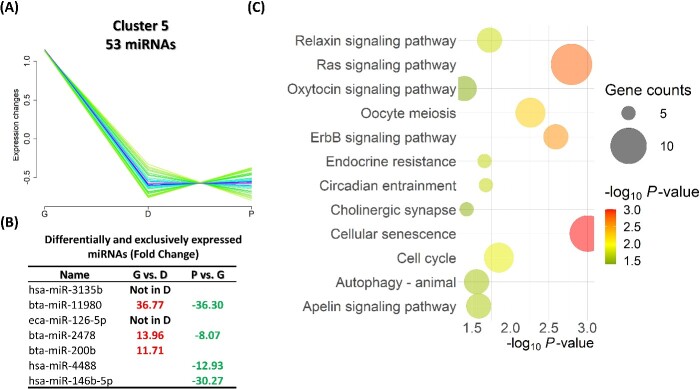
Cluster 5 consists of 53 miRNAs. (A) A group of 53 miRNAs exhibited a sharp reduction in their expression in the D and P stages, however, not in the G stage. (B) Among this cluster, seven miRNAs were differentially or exclusively expressed in different comparisons. (C) Pathways associated with the exclusive and DE-miRNA target genes of this cluster. G: growing; D: dominant; P: preovulatory.

**Figure 8:**
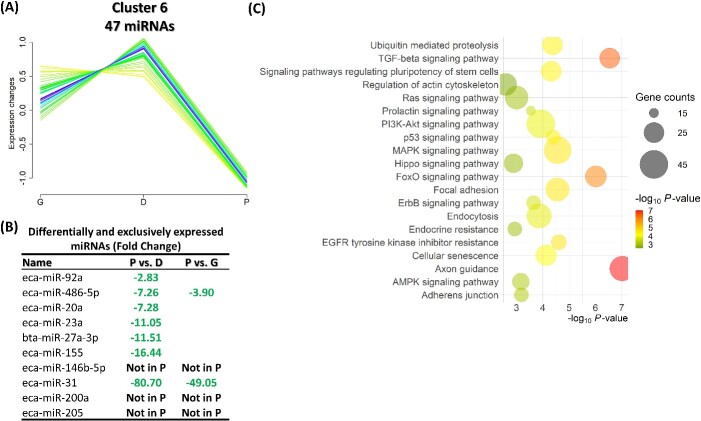
Cluster 6 consists of 47 miRNAs. (A) A group of 47 miRNAs exhibited a slight increase in their expression from the G to the D stage and then a sharp reduction at the P stage. (B) Among this cluster, 10 miRNAs were differentially or exclusively expressed in the different comparisons. (C) Pathways associated with the exclusive and DE-miRNA target genes of this cluster. G: growing; D: dominant; P: preovulatory.

Cluster 3 comprises a group of 67 miRNAs that exhibited an increase in their expression from the G until the D stage and then a reduction at the P stage (Figure 6A). Among this cluster, eight miRNAs, including eca-miR-148a, were differentially or exclusively expressed amidst the different groups (Figure 6B). Pathways associated with the exclusive and DE-miRNA target genes of this cluster exhibited that several signaling pathways (including RAS, P13K-Akt, P53, mTOR, and MAPK), focal adhesion, cellular senescence, and endocrine resistance were among the top significantly targeted pathways (Figure 6C). Cluster 5 comprises a group of 53 miRNAs that exhibited a sharp reduction in their expression in the D and P stages but not in the G stage (Figure 7A). Among this cluster, seven miRNAs were differentially or exclusively expressed amidst the different groups, including bta-miR-11980 with approximately a 36-fold increase in the G groups compared to both the D and P groups (Figure 7B). Pathways associated with the exclusive and DE-miRNA target genes of this cluster showed that oocyte meiosis, cellular senescence, and cell cycle were among the top significantly targeted pathways (Figure 7C). Cluster 6 comprises a group of 47 miRNAs that exhibited a high expression level at the G and D stages and then a sharp reduction at the P stage. Among this cluster, 10 miRNAs, including eca-miR-486-5p and eca-miR-31, were differentially or exclusively expressed amidst the different comparisons. Pathways associated with the exclusive and DE-miRNA target genes of this cluster showed similar pathways as in cluster 3, including several signaling pathways and endocrine resistance (Figure 8C).

## Discussion

Recent achievements in ARTs in the white rhinoceros [1, 16–18] have elucidated numerous aspects of reproduction and improved *in vitro* technologies for this species. Ovum pick up, *in vitro* oocyte maturation, fertilization, and embryo culture, as well as embryo transfer [1] have been implemented in the white rhinoceros, providing hope for the successful restoration of the genetic diversity necessary for a self-sustainable population. However, with very few scientific groups working on this challenging task worldwide, innovative approaches to increase the efficiency of *in vitro* technologies are imperative. Molecular mechanisms of follicle dynamics and ovarian function are still unmapped in the rhinoceros, hence the need to develop more sophisticated tools used in other model species.

At their core, EVs establish and liaise the molecular dialog between the growing oocyte and maternal tissues, through which miRNAs are the essential elements regulating follicular maturation [46, 47]. Previous studies from our group have implicated the role of EV-coupled miRNAs from FF in mediating the strictly coordinated and intricate crosstalk of follicular cells to ultimately ensure the ovulation of a competent oocyte [21]. Given that EVs [31] containing miRNAs [48] are readily uptaken by growing oocytes and embryos, and that FF-EVs categorically have been shown to stimulate cumulus expansion (morphologic and via gene regulation) *in vitro* [49], the aim of this study encompasses elucidating the dynamics and potential roles of EV-miRNAs from stage-specific FF amidst follicle growth for the first time in the SWR.

The presented data identify deviations among groups in miRNA expression levels from FF-EVs implying the probable regulatory machinery affecting the developing oocyte and the circumambient microenvironment. Due to the nature and rarity of the sample collections, to our knowledge, this is the first comparative analysis of FF-EVs and miRNAs, aimed toward a better understanding of the reproductive complexities and the prospective preservation of the rhinoceros species through potential improvements in ART efficiencies. Although the differences in individual animals were the first-factor variable removed, the PCA of quantified miRNAs clearly segments group D from P, which are more defined and structured in stage, while the fate of group G remains undifferentiated at the time of collection. This is a clear indication revealing that the EV-coupled miRNA fingerprint within the rhinoceros FF is directly associated with their follicular differentiation. Overall, a total of 231 miRNAs (55.4%) were identified to be commonly expressed among the three groups, while 34 (8.2%), 23 (5.5%), and 32 (7.7%), represent those exclusively expressed within the G, D, and P groups, respectively. These data suggest prominent alterations in the EV-miRNA cargo during different stages of follicle development, in accordance with previous work in bovine [21], humans [46], goats [50], and equine [24].

### Functional implications of miRNA expression and DE-miRNAs during segmented early stages of follicular development

Amidst the top highly expressed miRNAs, based on read counts, from the top 20 most abundant miRNAs (Table 1) in all groups include, eca-miR-148a, bta-miR-451, eca-miR-21, and bta-let-7i. To this point, the abundant expression patterns of eca-miR-148a and eca-miR-21, harmoniously follow suit with our previous publication of FF-EVs from stage-specific follicles in the mare, an adjacent representative model for reproduction in the rhinoceros [24]. Furthermore, a similar approach using FF-EVs in humans demonstrated the correlation of hsa-miR-148a with Day-3 embryo quality [51], and miR-21 in mouse oocyte maturation, blastocyst formation, and pre-implantation embryo development [52]. Analogously, the low expression of miR-451 in FF from endometriosis patients has been suggested to significantly affect oocyte competence for fertilization and pre-implantation embryo development via the aberrant expression of genes regulating the WNT signaling pathway [53]. Thus, it is likely that the integrative roles of these highly abundant EV-miRNAs may play critical function in regulating the timely undertaking needed to ovulate a competent gamete with the high-yielding capacity to develop past the blastocyst stage. Additionally, and consistent with our results, a previous study in cattle from different-sized follicles noted stable expression patterns of bta-let-7i in cumulus-oocyte complexes (COCs), denuded oocytes, and granulosa cells, albeit, a tendency for an increased expression of *DICER1* (miRNA processing machinery) in COCs from larger follicles [54]. These results suggest that during oogenesis, as follicles increase in size, the interaction between the oocyte and the surrounding follicular somatic cells may trigger miRNA machinery that directly involves the process of follicular maturation and ovulation. Further studies warrant the necessity to unveil the expression of such candidate miRNAs in biologically divergent samples collected from individual follicles based on size and subsequent maturation capacity *in vitro*.

Investigation of the effect of follicle size on the abundance of EV-miRNAs permitted the venture to transitionally decipher the alterations in small RNA cargoes governing follicle growth. The significant and exclusively expressed miRNAs in the D and G groups that don’t meet the expression threshold in the P group include eca-miR-146b-5p, eca-miR-200a, and eca-miR-205, as observed by the green arrows in Figure 3(C). Recent studies found that EVs collected from failed embryonic development (non-blastocyst) contained high expression of bta-miR-146b [48], while the use of synthetic bta-miR-146b mimics revealed its transcriptional regulatory role in inducing apoptosis and encumbering embryo development and quality [55]. On the other hand, the absence of miR-378 in the G group may be dually regulated by the precise stage of the developing follicle. MicroRNA-378 has been posed to regulate intrafollicular estradiol production by targeting aromatase (*CYP19A1*) during specific stages of follicular development [56–58]. In accordance, levels of FF estradiol are increased in larger follicles further participating in the selection of a dominant preovulatory follicle [59], whereas the fate of growing follicles may be undetermined to this point.

Numerous DE-miRNAs amidst the three comparisons (G vs. D, P vs. G, and P vs. D; Figure 4) involve critical regulatory roles in the potential to govern follicular growth. Case in point, bta-miR-200b and eca-miR-379 upregulated in the G vs. D group, have been previously shown to be essential for ovulation and fertility in mice [60, 61], while granulosa cell-secreted exosomal miR-379-5P was shown as a proliferative response to androgenic stimulation in preantral stage follicular development [62]. Taken together, these data suggest a firmly integrated association between the EV-miRNA intrafollicular response and follicle growth/size. MicroRNA-200c downregulated in the P vs. G comparison, aligns in accordance with a study in humans where diminished expression of hsa-miR-200c-5p positively correlated with embryos permitting successful pregnancy [63]. Also in this study, eca-miR-423-3p (up-), eca-miR-155 (down-), were DE (P vs. D), and have been shown to inhibit granulosa cell apoptosis [64] and negatively regulate cumulus cell function, oocyte maturation, and blastocyst formation [65]. Whether the aforementioned DE-miRNAs serve as potential biomarkers governing follicle status for ovulation of a competent gamete remains to be determined.

### miRNA cluster involvement via pathway regulation in developing follicles

The miRNA cluster analysis identified nine different expression patterns during the three follicular developmental stages. Three out of the nine clusters involved a higher number of DE-miRNAs. Clusters 3 and 6 shared a similar pattern, in which the miRNAs exhibited peak expression at the D stage compared to the G and P stages. Although both clusters include different DE-miRNAs, they showed similar targeted signaling pathways. Several studies have demonstrated the role of signaling pathways in coordinating ovarian follicle development [66]. During follicle development, primordial follicle activation and oocyte growth are under the control of PI3K/AKT/FOXO3 signaling pathways [67]. Targeting *PI3K* via the abundant expression of miR-214-3p disrupts PI3K/AKT/FOXO3 signaling pathways, leading to the inhibition of primordial follicle development [68]. Additionally, miRNAs are known to be involved in regulating the proliferation and apoptosis of granulosa cells [69, 70], and follicular development [71]. In bovine, a cluster of miRNAs, including miR-183-96-182, was among the top most significantly upregulated miRNAs in granulosa cells of dominant follicles compared to subordinate follicles [72]. In that study, we suggested that this miRNA cluster regulates and promotes granulosa cell proliferation by targeting the proapoptotic gene *FOXO1.* Similarly, in the current study, miR-183 was among cluster 3 with a significantly higher expression in the D compared to the P stage. Another interesting miRNA in cluster 6 is miR-92a, which exhibited the same pattern as miR-183. Previously, it has been reported that miR-92 coordinately regulates granulosa cell proliferation and differentiation by targeting *PTEN* and *BMPR2* genes [73]. The upregulation of miR-92 promoted granulosa cell proliferation, while its downregulation increased differentiation.

Cluster 5 exhibited a consistent declining expression pattern from the G toward the D and P stage groups. Interestingly, oocyte meiosis, cellular senescence, and cell cycle were among the top pathways targeted by the miRNAs of this cluster. In most mammals, it is well known that antral follicles maintain the first meiotic arrest of the oocytes during the diplotene stage of prophase I through the elevation of the intracellular cAMP levels that inactivate the oocyte maturation-promoting factor [74]. Additionally, several studies have demonstrated the potential role of miRNAs in regulating oocyte meiosis [75]. In yak oocytes, miR-342-3p was reported to play an important role in meiotic maturation by targeting *DNMT1* [76]. The same miRNA is involved in different biological processes to include inducing cancer cell proliferation arrest [77, 78]. Moreover, the role of miRNAs in regulating several biological functions in granulosa cells including cell cycle and senescence has been reported [79]. Among these miRNAs, miR-200b, one of the DE-miRNAs in cluster 5, was recently reported to suppress proliferation and induce senescence in ovine granulosa cells [80], indicating its role in follicular atresia. This may explain the pattern of this miRNA cluster with a higher abundance during the G phase, in which follicular atresia will take place, compared to the D and P phases. These findings together with our results, indicate the potential crucial role of EV-coupled miRNAs in folliculogenesis, atresia, and oocyte maturation in the rhinoceros, as it has been reported in various other domestic species [38, 71, 81]. Comprehensive studies on the precise roles of miRNAs in ovarian follicular development and oocyte meiosis will provide important insights into female reproductive fertility aiding to improving ARTs in this near-threatened species. Although *in vitro* maturation and culture systems have been developed for the SWR [1, 32], *in vitro* results remain largely suboptimal compared to domestic species, and improvements are necessary to have a large scale impact on the outcome of assisted reproduction. The possibility of using EVs to better understand EV-coupled miRNA dynamics regulating follicular growth to aide in improving the *in vitro* development of SWR embryos provides an uncharted and novel approach to support and facilitate conservation efforts in this species. Moreover, potential crossovers of EV-coupled molecular signaling during folliculogenesis in the SWR, could also provide valuable input to the identification of physiological and pathological reproductive conditions propagating abnormal fertility, and ultimately to the development of new reproductive strategies to improve ART outcomes for this species and many others.

## Supplementary Material

Supplementary_Files_04242024_ioae081

## Data Availability

Raw FASTQ files and the processed CSV files of the miRNA sequencing data were deposited in the National Center for Biotechnology Information (NCBI) Gene Expression Omnibus (GEO) database (https://www.ncbi.nlm.nih.gov/geo/) and can be accessed with the accession number GSE246260. Reported details on the experimental procedures concerning EVs have been submitted to the EV-TRACK knowledgebase with open access under (EV-TRACK ID: EV240017). Additional information regarding the findings of this study is available upon request from the corresponding author.
