# Supplementary material for: Mapping the follicle-specific regulation of extracellular vesicle-mediated microRNA transport in the southern white rhinoceros (Ceratotherium simum simum)
Source: Biol Reprod. 2024 May 22;111(2):376–90. doi: 10.1093/biolre/ioae081 (PMC11327318; doi:10.1093/biolre/ioae081)
Supplement: Supplementary_Files_04242024_ioae081 [file supplementary_files_04242024_ioae081.docx]

| **Table S1: Summary of sequence reads mapped to the equine reference genome and annotated against equine miRNAs listed in the mirBase database.** | | | | | |  |  |  |  |  |  |  |  |  |
| --- | --- | --- | --- | --- | --- | --- | --- | --- | --- | --- | --- | --- | --- | --- |
| Group | Sample | Total number of reads | QC reads | % QC reads | % miRNA* reads |  |  |  |  |  |  |  |  |  |
| **Growing** | **G1** | 21,365,519 | 18,060,716 | 84.5 | 0.44 |  |  |  |  |  |  |  |  |  |
|  | **G2** | 20,767,820 | 14,761,271 | 71.1 | 0.17 |  |  |  |  |  |  |  |  |  |
|  | **G3** | 82,742,319 | 28,066,350 | 33.9 | 0.57 |  |  |  |  |  |  |  |  |  |
| **Dominant** | **D1** | 24,207,860 | 17,961,348 | 74.2 | 0.28 |  |  |  |  |  |  |  |  |  |
|  | **D2** | 20,872,978 | 15,484,328 | 74.2 | 1.05 |  |  |  |  |  |  |  |  |  |
|  | **D3** | 20,038,269 | 16,968,145 | 84.7 | 0.32 |  |  |  |  |  |  |  |  |  |
| **Pre-ovulatory** | **P1** | 20,913,752 | 17,150,844 | 82.0 | 0.38 |  |  |  |  |  |  |  |  |  |
|  | **P2** | 21,232,533 | 18,326,443 | 86.3 | 0.14 |  |  |  |  |  |  |  |  |  |
|  | **P3** | 18,685,590 | 16,312,070 | 87.3 | 0.26 |  |  |  |  |  |  |  |  |  |
| *The proportion of mapped reads annotated in the miRbase database | | | | | | |  |  |  |  |  |  |  |  |

| **Table S2: A complete list of all expressed miRNAs indicated as the mean of TMM-adjusted Counts Per Million (CPM) value** | | | | | | | |
| --- | --- | --- | --- | --- | --- | --- | --- |
| **Name** | **G** |  | **Name** | **D** |  | **Name** | **P** |
| bta-miR-11980 | 185839.3 |  | eca-miR-148a | 872829.2 |  | eca-miR-148a | 94882.4 |
| eca-miR-148a | 85922.5 |  | bta-miR-451 | 96984.3 |  | hsa-miR-10400-5p | 54664.7 |
| bta-miR-451 | 83159.5 |  | hsa-miR-10400-5p | 91187.3 |  | bta-miR-451 | 38476.7 |
| bta-miR-2478 | 50026.8 |  | eca-miR-21 | 45580.3 |  | bta-miR-99a-5p | 38245.9 |
| hsa-miR-3168 | 47238.0 |  | eca-miR-122 | 32587.9 |  | eca-miR-99a | 30796.4 |
| eca-miR-21 | 34231.7 |  | eca-miR-486-5p | 30169.5 |  | bta-let-7i | 24071.3 |
| bta-let-7i | 29962.8 |  | bta-let-7i | 28037.4 |  | eca-miR-21 | 23430.8 |
| hsa-miR-10400-5p | 24243.7 |  | hsa-miR-3168 | 26703.2 |  | eca-miR-10b | 21032.4 |
| bta-miR-99a-5p | 24046.2 |  | bta-miR-99a-5p | 23336.0 |  | eca-miR-143 | 20767.1 |
| eca-miR-26a | 20774.9 |  | eca-miR-26a | 21300.4 |  | eca-miR-26a | 18064.7 |
| eca-miR-1 | 20262.7 |  | bta-miR-192 | 17449.7 |  | bta-let-7b | 17731.9 |
| eca-let-7g | 16584.2 |  | eca-let-7g | 14136.2 |  | eca-let-7a | 16031.3 |
| eca-let-7f | 16230.6 |  | eca-miR-423-5p | 13883.4 |  | bta-miR-192 | 15927.3 |
| eca-miR-486-5p | 16206.6 |  | bta-let-7b | 13142.8 |  | eca-miR-122 | 15727.2 |
| bta-miR-151-3p | 15349.3 |  | eca-miR-126-3p | 12884.6 |  | eca-miR-100 | 15330.0 |
| eca-miR-143 | 13270.9 |  | eca-miR-143 | 11875.7 |  | eca-miR-1 | 13948.5 |
| eca-miR-423-5p | 12834.6 |  | eca-let-7f | 11162.9 |  | eca-miR-128 | 12321.1 |
| bta-let-7b | 12829.2 |  | eca-miR-128 | 10955.8 |  | eca-let-7f | 12199.4 |
| eca-miR-122 | 12587.2 |  | bta-miR-151-3p | 10827.0 |  | bta-miR-151-3p | 11359.7 |
| eca-miR-100 | 11684.0 |  | eca-miR-10b | 10642.1 |  | eca-miR-423-5p | 9912.5 |
| eca-miR-9a | 11637.2 |  | bta-miR-21-5p | 10099.6 |  | hsa-miR-3168 | 9857.5 |
| eca-let-7a | 10694.9 |  | eca-miR-1 | 9776.5 |  | eca-let-7g | 8869.9 |
| eca-miR-128 | 9567.6 |  | hsa-miR-4497 | 9102.4 |  | eca-miR-184 | 7888.8 |
| bta-miR-21-5p | 8497.6 |  | eca-miR-92a | 8298.4 |  | eca-miR-9a | 7415.3 |
| eca-miR-126-3p | 8457.5 |  | eca-miR-192 | 8203.6 |  | eca-let-7c | 7399.2 |
| eca-miR-27b | 8224.0 |  | eca-miR-100 | 8176.3 |  | bta-miR-21-5p | 6754.4 |
| eca-miR-10a | 8072.5 |  | bta-miR-320a | 7870.2 |  | bta-miR-143 | 6399.3 |
| eca-miR-99a | 7633.6 |  | eca-miR-99a | 7520.9 |  | bta-miR-9-5p | 6235.0 |
| eca-miR-10b | 7354.2 |  | bta-miR-215 | 6952.2 |  | bta-miR-215 | 6214.0 |
| eca-miR-92a | 7139.3 |  | eca-miR-27b | 6579.9 |  | bta-miR-2478 | 6198.1 |
| bta-miR-9-5p | 6991.5 |  | bta-miR-1246 | 6510.1 |  | bta-miR-320a | 5902.5 |
| eca-let-7c | 6840.9 |  | hsa-miR-4492 | 6437.5 |  | hsa-miR-574-5p | 5488.8 |
| eca-miR-30d | 6580.5 |  | bta-miR-378 | 6296.5 |  | eca-miR-192 | 5480.3 |
| eca-miR-200b | 5597.8 |  | eca-let-7a | 6053.9 |  | bta-miR-11980 | 5117.0 |
| bta-miR-192 | 5588.2 |  | bta-miR-11980 | 5051.4 |  | eca-miR-126-3p | 5076.7 |
| eca-miR-192 | 5547.3 |  | eca-let-7c | 4887.6 |  | bta-miR-1246 | 4914.7 |
| eca-miR-184 | 5513.7 |  | eca-miR-148b-3p | 4023.2 |  | eca-miR-127 | 4394.7 |
| bta-miR-143 | 5302.9 |  | eca-miR-30d | 3915.9 |  | hsa-miR-4497 | 4275.7 |
| hsa-miR-4488 | 4393.7 |  | bta-miR-7 | 3712.2 |  | eca-miR-10a | 4265.3 |
| eca-miR-127 | 4359.0 |  | eca-miR-10a | 3657.5 |  | eca-miR-486-5p | 4144.7 |
| hsa-miR-30a-5p | 4083.9 |  | bta-miR-2478 | 3580.6 |  | bta-miR-378 | 3886.7 |
| bta-miR-1246 | 3928.4 |  | eca-miR-27a | 3508.6 |  | bta-miR-7 | 3831.3 |
| bta-miR-320a | 3663.5 |  | eca-miR-25 | 3020.8 |  | eca-miR-30d | 3799.9 |
| eca-miR-30c | 3639.0 |  | eca-miR-24 | 3015.4 |  | eca-miR-27b | 3645.6 |
| bta-miR-378 | 3508.1 |  | eca-miR-200b | 2960.5 |  | eca-miR-206 | 3620.1 |
| bta-miR-30d | 3314.2 |  | eca-miR-22 | 2785.0 |  | eca-miR-125b-5p | 3580.0 |
| eca-miR-99b | 3312.9 |  | bta-miR-11987 | 2675.5 |  | eca-miR-423-3p | 3522.4 |
| bta-miR-11987 | 3290.4 |  | eca-miR-191a | 2543.0 |  | eca-miR-125a-5p | 3493.7 |
| bta-miR-200b | 3220.1 |  | bta-miR-30d | 2435.2 |  | eca-miR-199a-3p | 2987.8 |
| eca-miR-101 | 3042.1 |  | hsa-miR-3195 | 2420.8 |  | eca-miR-92a | 2940.4 |
| eca-miR-25 | 2925.1 |  | bta-miR-27a-3p | 2419.9 |  | bta-miR-30d | 2715.7 |
| eca-miR-423-3p | 2834.5 |  | eca-miR-200a | 2334.9 |  | bta-miR-125a | 2629.5 |
| eca-miR-221 | 2783.5 |  | eca-miR-101 | 2332.2 |  | bta-miR-11987 | 2505.7 |
| bta-miR-7 | 2717.5 |  | eca-miR-30c | 2215.5 |  | hsa-miR-4492 | 2481.9 |
| eca-miR-191a | 2716.0 |  | bta-miR-143 | 2157.7 |  | eca-miR-99b | 2448.2 |
| eca-miR-125b-5p | 2639.0 |  | eca-miR-221 | 2134.4 |  | hsa-miR-3960 | 2217.8 |
| eca-miR-24 | 2616.5 |  | eca-miR-146a | 2075.3 |  | eca-miR-30c | 2215.6 |
| eca-miR-125a-5p | 2616.4 |  | eca-miR-99b | 2065.4 |  | eca-miR-215 | 2054.1 |
| eca-miR-107a | 2507.7 |  | eca-miR-127 | 1894.0 |  | eca-miR-191a | 1980.2 |
| eca-miR-181a | 2364.3 |  | hsa-miR-574-5p | 1868.5 |  | eca-miR-24 | 1954.3 |
| hsa-miR-574-5p | 2211.5 |  | hsa-miR-3960 | 1757.9 |  | eca-miR-200b | 1918.5 |
| bta-miR-30a-5p | 2109.2 |  | eca-miR-125b-5p | 1723.6 |  | eca-miR-101 | 1740.4 |
| eca-miR-199a-3p | 2102.1 |  | bta-miR-30a-5p | 1664.0 |  | bta-miR-22-3p | 1697.0 |
| eca-miR-499-5p | 1992.7 |  | bta-miR-142-5p | 1632.7 |  | eca-let-7e | 1693.7 |
| eca-miR-27a | 1558.9 |  | eca-miR-205 | 1608.7 |  | eca-miR-221 | 1606.1 |
| eca-miR-200c | 1512.8 |  | hsa-miR-223-5p | 1602.5 |  | eca-miR-25 | 1517.5 |
| bta-miR-215 | 1480.7 |  | eca-miR-181a | 1549.5 |  | eca-miR-181a | 1499.0 |
| eca-miR-194 | 1474.8 |  | eca-miR-31 | 1537.5 |  | eca-miR-199a-5p | 1464.7 |
| eca-miR-92b | 1433.6 |  | eca-miR-107a | 1526.1 |  | bta-miR-202 | 1447.7 |
| eca-miR-200a | 1430.6 |  | bta-miR-202 | 1488.6 |  | eca-miR-7 | 1380.0 |
| bta-miR-27a-3p | 1414.9 |  | eca-miR-199a-3p | 1425.8 |  | bta-miR-455-5p | 1226.4 |
| bta-miR-125a | 1377.0 |  | bta-miR-140 | 1266.1 |  | eca-miR-148b-3p | 1209.7 |
| eca-let-7d | 1372.2 |  | bta-miR-185 | 1262.3 |  | hsa-miR-7704 | 1199.3 |
| hsa-miR-146b-5p | 1364.3 |  | hsa-miR-361-3p | 1245.8 |  | eca-miR-107a | 1183.6 |
| eca-let-7e | 1345.6 |  | eca-miR-23a | 1239.3 |  | hsa-miR-3195 | 1164.4 |
| eca-miR-129a-5p | 1339.0 |  | hsa-miR-155-5p | 1215.5 |  | hsa-miR-30a-5p | 1163.2 |
| bta-miR-202 | 1288.4 |  | eca-miR-146b-5p | 1211.7 |  | bta-miR-182 | 1075.0 |
| eca-miR-205 | 1179.2 |  | eca-miR-486-3p | 1204.4 |  | eca-miR-381 | 1046.6 |
| bta-miR-140 | 1178.6 |  | eca-miR-125a-5p | 1173.1 |  | eca-miR-27a | 1041.1 |
| hsa-miR-3960 | 1162.0 |  | bta-miR-744 | 1158.9 |  | bta-miR-221 | 1020.8 |
| eca-miR-199a-5p | 1100.5 |  | eca-miR-499-5p | 1148.7 |  | bta-miR-140 | 948.4 |
| eca-miR-148b-3p | 1072.2 |  | eca-miR-183 | 1101.6 |  | eca-miR-199b-5p | 920.2 |
| eca-miR-186 | 1070.0 |  | eca-miR-155 | 1085.4 |  | bta-miR-30e-5p | 893.3 |
| hsa-miR-3135b | 1033.0 |  | eca-miR-451 | 1004.2 |  | bta-miR-152 | 885.9 |
| bta-miR-22-3p | 1017.6 |  | eca-miR-93 | 1000.2 |  | hsa-miR-200a-5p | 861.6 |
| eca-miR-615-3p | 1006.2 |  | bta-miR-26b | 922.2 |  | eca-miR-222 | 842.4 |
| eca-miR-124 | 981.9 |  | eca-miR-20a | 918.3 |  | eca-miR-615-3p | 829.1 |
| eca-miR-31 | 939.7 |  | hsa-miR-30a-5p | 917.2 |  | bta-miR-493 | 827.0 |
| hsa-miR-615-3p | 934.1 |  | hsa-miR-4488 | 915.1 |  | eca-miR-194 | 825.0 |
| eca-miR-328 | 915.8 |  | eca-miR-98 | 897.4 |  | bta-miR-744 | 806.1 |
| eca-miR-381 | 875.8 |  | hsa-miR-4508 | 885.1 |  | bta-miR-30a-5p | 785.3 |
| bta-miR-26b | 861.0 |  | eca-miR-215 | 858.6 |  | hsa-miR-451a | 755.7 |
| eca-miR-7 | 845.4 |  | eca-miR-532-5p | 834.1 |  | eca-miR-22 | 704.1 |
| eca-miR-146a | 793.3 |  | eca-miR-423-3p | 830.0 |  | eca-miR-370 | 704.0 |
| eca-miR-29a | 769.3 |  | eca-miR-9a | 812.9 |  | hsa-miR-516b-5p | 684.7 |
| eca-miR-370 | 746.6 |  | eca-miR-328 | 792.9 |  | bta-miR-375 | 680.4 |
| hsa-miR-7704 | 740.5 |  | eca-miR-7 | 788.2 |  | eca-miR-1307 | 666.4 |
| bta-miR-30e-5p | 722.9 |  | eca-miR-218 | 770.8 |  | eca-miR-196a | 664.1 |
| eca-miR-23a | 706.1 |  | hsa-miR-7704 | 749.2 |  | bta-miR-200b | 640.1 |
| hsa-miR-375-3p | 688.1 |  | hsa-miR-452-5p | 748.8 |  | eca-miR-532-5p | 636.4 |
| eca-miR-20a | 681.6 |  | hsa-miR-203a-3p | 748.8 |  | hsa-miR-576-3p | 635.5 |
| eca-miR-155 | 657.0 |  | bta-miR-22-3p | 739.0 |  | eca-miR-328 | 631.3 |
| eca-miR-206 | 646.7 |  | hsa-miR-375-3p | 706.6 |  | bta-miR-199a-5p | 601.6 |
| hsa-miR-203a-3p | 644.6 |  | bta-miR-221 | 703.8 |  | bta-miR-2428 | 586.8 |
| bta-miR-29a | 640.3 |  | eca-miR-200c | 683.9 |  | hsa-miR-320c | 570.3 |
| eca-miR-28-3p | 632.2 |  | eca-miR-150 | 675.2 |  | hsa-let-7d-3p | 569.0 |
| hsa-miR-4497 | 625.9 |  | bta-miR-146b | 656.8 |  | hsa-miR-375-3p | 563.0 |
| hsa-miR-4492 | 615.0 |  | bta-miR-125a | 642.1 |  | eca-let-7d | 556.1 |
| bta-miR-744 | 606.1 |  | eca-miR-1839 | 633.5 |  | hsa-miR-223-5p | 503.5 |
| eca-miR-204b | 602.9 |  | hsa-miR-320c | 616.8 |  | eca-miR-486-3p | 490.3 |
| eca-miR-486-3p | 599.0 |  | hsa-miR-1290 | 594.4 |  | eca-miR-129a-5p | 483.4 |
| eca-miR-22 | 585.8 |  | eca-miR-199a-5p | 581.5 |  | bta-miR-574 | 470.0 |
| bta-miR-221 | 580.7 |  | eca-miR-378 | 524.3 |  | bta-let-7a-3p | 469.4 |
| eca-miR-379 | 573.2 |  | eca-miR-17 | 470.2 |  | eca-miR-186 | 461.8 |
| hsa-miR-3195 | 565.1 |  | hsa-miR-106b-3p | 451.3 |  | eca-miR-204b | 451.4 |
| bta-miR-142-5p | 562.8 |  | bta-miR-30e-5p | 434.0 |  | bta-miR-185 | 445.3 |
| bta-miR-185 | 552.7 |  | bta-miR-2428 | 433.6 |  | hsa-miR-615-3p | 445.0 |
| hsa-miR-451a | 530.8 |  | hsa-miR-3615 | 433.6 |  | eca-miR-30b | 443.1 |
| eca-miR-145 | 529.8 |  | eca-miR-186 | 423.7 |  | eca-miR-499-5p | 420.3 |
| eca-miR-146b-5p | 510.8 |  | bta-miR-9-5p | 419.7 |  | eca-miR-124 | 412.6 |
| hsa-miR-361-3p | 499.7 |  | eca-miR-129a-5p | 417.2 |  | eca-miR-409-3p | 383.5 |
| hsa-miR-30e-3p | 482.4 |  | eca-let-7d | 416.3 |  | hsa-miR-4508 | 368.8 |
| hsa-miR-9-3p | 481.7 |  | hsa-miR-335-3p | 415.3 |  | eca-miR-150 | 367.7 |
| eca-miR-363 | 469.2 |  | hsa-let-7d-3p | 408.7 |  | hsa-miR-26b-5p | 352.1 |
| hsa-miR-345-5p | 453.8 |  | bta-miR-139 | 397.7 |  | eca-miR-379 | 349.3 |
| eca-miR-218 | 450.2 |  | eca-miR-1307 | 397.0 |  | eca-miR-17 | 343.3 |
| bta-miR-152 | 447.8 |  | eca-miR-29a | 396.5 |  | eca-miR-145 | 341.9 |
| bta-miR-146b | 440.3 |  | eca-miR-222 | 390.0 |  | hsa-miR-4488 | 337.4 |
| bta-miR-1260b | 429.0 |  | hsa-miR-30e-3p | 383.3 |  | eca-miR-92b | 330.8 |
| hsa-miR-26b-5p | 425.5 |  | eca-miR-28-3p | 378.4 |  | eca-miR-197 | 330.5 |
| bta-miR-423-3p | 417.9 |  | hsa-miR-125b-1-3p | 376.8 |  | hsa-miR-125b-2-3p | 329.6 |
| hsa-miR-320c | 413.2 |  | eca-miR-506a | 368.6 |  | hsa-miR-210-3p | 328.6 |
| eca-miR-23b | 412.5 |  | eca-let-7e | 344.5 |  | hsa-miR-361-3p | 327.1 |
| hsa-let-7d-3p | 411.2 |  | hsa-miR-451a | 334.5 |  | hsa-miR-493-5p | 321.5 |
| bta-miR-455-5p | 410.6 |  | eca-miR-363 | 316.3 |  | bta-miR-200a | 295.7 |
| bta-miR-182 | 395.0 |  | hsa-miR-30a-3p | 308.5 |  | bta-miR-126-3p | 289.5 |
| bta-miR-1307 | 390.2 |  | eca-miR-142-3p | 304.9 |  | eca-miR-181b | 288.8 |
| hsa-miR-30a-3p | 386.7 |  | hsa-miR-214-5p | 303.3 |  | eca-miR-133a | 285.9 |
| bta-miR-23a | 379.1 |  | bta-miR-23a | 295.6 |  | eca-miR-149 | 279.0 |
| hsa-miR-4508 | 372.6 |  | hsa-miR-146b-5p | 290.5 |  | bta-miR-484 | 278.6 |
| eca-miR-222 | 370.5 |  | eca-miR-92b | 276.2 |  | bta-miR-23b-3p | 273.9 |
| bta-miR-23b-3p | 370.2 |  | bta-miR-182 | 276.1 |  | bta-miR-27a-5p | 271.8 |
| bta-miR-200a | 367.7 |  | bta-miR-200b | 273.2 |  | hsa-miR-1290 | 248.4 |
| eca-miR-199b-5p | 366.0 |  | bta-miR-29a | 242.2 |  | eca-miR-218 | 246.9 |
| eca-miR-1298 | 364.7 |  | hsa-miR-331-5p | 241.1 |  | eca-miR-146a | 246.7 |
| eca-miR-126-5p | 356.1 |  | eca-miR-23b | 236.9 |  | bta-miR-26b | 243.8 |
| eca-miR-340-5p | 347.9 |  | hsa-miR-941 | 235.4 |  | hsa-miR-30a-3p | 236.1 |
| eca-miR-150 | 345.7 |  | eca-miR-370 | 233.4 |  | hsa-miR-200b-5p | 233.1 |
| hsa-miR-106b-3p | 344.3 |  | eca-miR-197 | 228.1 |  | hsa-miR-331-5p | 232.6 |
| hsa-miR-331-5p | 339.0 |  | eca-miR-433 | 218.3 |  | hsa-miR-378d | 231.0 |
| eca-miR-93 | 337.1 |  | eca-miR-194 | 210.6 |  | bta-miR-423-3p | 224.6 |
| eca-miR-30b | 313.4 |  | bta-miR-152 | 205.8 |  | hsa-miR-125b-1-3p | 214.8 |
| hsa-miR-1843 | 311.5 |  | eca-miR-149 | 204.9 |  | eca-miR-16 | 214.5 |
| bta-miR-375 | 310.5 |  | eca-miR-340-5p | 202.5 |  | hsa-miR-30e-3p | 213.6 |
| hsa-miR-210-3p | 308.6 |  | bta-miR-760-3p | 200.2 |  | eca-miR-28-3p | 210.4 |
| hsa-miR-320b | 305.8 |  | eca-miR-30b | 198.6 |  | bta-miR-27a-3p | 208.4 |
| hsa-miR-24-2-5p | 304.9 |  | eca-miR-140-5p | 198.4 |  | hsa-miR-9-3p | 201.3 |
| bta-miR-425-5p | 303.8 |  | bta-miR-425-5p | 184.8 |  | eca-miR-671-5p | 199.7 |
| eca-miR-181b | 298.1 |  | bta-miR-17-5p | 180.6 |  | hsa-miR-152-3p | 196.6 |
| eca-miR-532-5p | 296.4 |  | eca-miR-181b | 177.5 |  | eca-miR-433 | 183.3 |
| hsa-miR-4443 | 278.1 |  | bta-miR-330 | 160.6 |  | eca-miR-95 | 182.8 |
| bta-miR-139 | 272.7 |  | bta-miR-27a-5p | 158.5 |  | eca-miR-671-3p | 175.7 |
| eca-miR-16 | 267.6 |  | eca-miR-140-3p | 153.7 |  | eca-miR-141 | 175.1 |
| eca-miR-598 | 257.4 |  | hsa-miR-24-2-5p | 149.8 |  | eca-miR-330 | 175.1 |
| eca-miR-744 | 257.2 |  | bta-miR-183 | 144.2 |  | hsa-miR-145-3p | 165.8 |
| hsa-miR-335-3p | 250.4 |  | bta-miR-1307 | 142.9 |  | bta-miR-29a | 159.9 |
| hsa-miR-223-5p | 248.7 |  | eca-miR-132 | 141.5 |  | eca-miR-98 | 153.0 |
| eca-miR-133a | 247.3 |  | eca-miR-330 | 138.5 |  | eca-miR-96 | 152.3 |
| hsa-miR-574-3p | 235.4 |  | hsa-miR-200a-5p | 134.0 |  | eca-miR-183 | 149.5 |
| hsa-miR-219a-2-3p | 235.3 |  | bta-miR-455-3p | 133.0 |  | bta-miR-10174-3p | 145.9 |
| eca-miR-17 | 231.7 |  | bta-miR-375 | 130.3 |  | eca-miR-20a | 135.6 |
| eca-miR-409-3p | 227.2 |  | hsa-miR-320d | 124.5 |  | eca-miR-132 | 134.0 |
| eca-miR-34c | 226.5 |  | hsa-miR-26b-5p | 123.2 |  | eca-miR-340-5p | 131.1 |
| eca-miR-330 | 226.4 |  | eca-miR-381 | 119.2 |  | bta-miR-330 | 130.9 |
| bta-miR-330 | 226.2 |  | eca-miR-124 | 112.9 |  | eca-miR-93 | 126.5 |
| bta-miR-411a | 221.1 |  | bta-miR-11975 | 111.8 |  | eca-miR-378 | 125.1 |
| eca-miR-183 | 215.5 |  | eca-miR-342-3p | 110.2 |  | eca-miR-450c | 123.5 |
| bta-miR-129 | 208.3 |  | eca-miR-16 | 109.7 |  | eca-miR-134 | 122.5 |
| eca-miR-98 | 205.8 |  | eca-miR-450a | 108.7 |  | bta-miR-181d | 120.1 |
| bta-miR-27a-5p | 199.0 |  | bta-miR-23b-3p | 107.6 |  | eca-miR-323-3p | 117.4 |
| bta-miR-484 | 188.2 |  | eca-miR-340-3p | 104.7 |  | eca-miR-411 | 117.1 |
| bta-miR-493 | 180.3 |  | bta-miR-1260b | 93.7 |  | eca-miR-543 | 116.8 |
| eca-miR-140-3p | 180.0 |  | bta-let-7e | 91.4 |  | eca-miR-23a | 114.5 |
| eca-miR-433 | 179.9 |  | eca-miR-142-5p | 91.1 |  | eca-miR-450b-5p | 114.1 |
| eca-miR-134 | 177.1 |  | bta-miR-484 | 88.5 |  | eca-miR-29a | 111.4 |
| hsa-miR-195-3p | 173.3 |  | bta-miR-200a | 87.6 |  | hsa-miR-106b-3p | 110.2 |
| eca-miR-142-3p | 172.6 |  | hsa-miR-3158-3p | 85.2 |  | eca-miR-23b | 109.8 |
| eca-miR-411 | 167.2 |  | bta-miR-423-3p | 82.7 |  | hsa-miR-941 | 103.9 |
| eca-miR-1839 | 165.2 |  | eca-miR-223 | 81.3 |  | eca-miR-542-3p | 103.8 |
| hsa-miR-493-5p | 161.8 |  | eca-miR-15b | 81.3 |  | hsa-miR-127-5p | 101.4 |
| bta-miR-126-3p | 161.4 |  | hsa-miR-21-3p | 77.4 |  | hsa-miR-335-3p | 100.8 |
| eca-miR-196a | 158.3 |  | eca-miR-409-3p | 76.7 |  | bta-let-7e | 99.0 |
| bta-miR-10174-3p | 155.7 |  | hsa-miR-181a-3p | 70.4 |  | hsa-miR-99b-3p | 98.6 |
| eca-miR-193a-5p | 151.2 |  | bta-let-7a-3p | 69.5 |  | eca-miR-382 | 95.0 |
| bta-miR-17-5p | 143.8 |  | hsa-miR-219a-2-3p | 69.3 |  | eca-miR-451 | 93.7 |
| hsa-miR-127-5p | 143.7 |  | eca-miR-543 | 68.8 |  | eca-miR-374b | 91.3 |
| bta-let-7e | 143.7 |  | eca-miR-374b | 68.1 |  | hsa-miR-514a-3p | 89.8 |
| eca-miR-374b | 135.8 |  | hsa-miR-429 | 66.5 |  | hsa-miR-320d | 89.6 |
| hsa-miR-941 | 135.1 |  | hsa-miR-152-3p | 66.0 |  | bta-miR-2881 | 88.6 |
| eca-miR-451 | 133.0 |  | eca-miR-28-5p | 62.2 |  | eca-miR-140-3p | 87.4 |
| eca-miR-214 | 129.1 |  | hsa-miR-629-5p | 60.7 |  | bta-miR-411a | 86.4 |
| eca-miR-382 | 126.3 |  | bta-miR-11976 | 58.8 |  | eca-miR-652 | 85.3 |
| eca-miR-223 | 123.0 |  | eca-miR-106b | 56.5 |  | hsa-miR-181a-2-3p | 85.3 |
| hsa-miR-152-3p | 123.0 |  | eca-miR-382 | 56.0 |  | hsa-miR-221-5p | 84.8 |
| eca-miR-1307 | 119.9 |  | eca-miR-129b-3p | 53.7 |  | hsa-miR-1843 | 82.5 |
| eca-miR-215 | 119.8 |  | eca-miR-199b-5p | 53.2 |  | bta-miR-139 | 81.9 |
| eca-miR-342-3p | 119.6 |  | hsa-miR-675-5p | 53.0 |  | hsa-miR-4516 | 81.8 |
| hsa-miR-181a-2-3p | 118.4 |  | eca-miR-485-5p | 50.2 |  | eca-miR-30e | 81.7 |
| hsa-miR-125b-1-3p | 116.8 |  | bta-miR-181b | 49.7 |  | bta-miR-339a | 78.6 |
| eca-miR-506a | 116.3 |  | hsa-miR-181a-2-3p | 48.3 |  | bta-miR-23a | 77.8 |
| hsa-miR-4516 | 116.2 |  | eca-miR-182 | 47.4 |  | eca-miR-34c | 76.7 |
| eca-miR-224 | 110.2 |  | hsa-miR-139-3p | 45.5 |  | bta-miR-425-5p | 74.1 |
| eca-miR-149 | 108.9 |  | eca-miR-379 | 45.1 |  | eca-miR-200c | 73.9 |
| eca-miR-323-3p | 104.3 |  | eca-miR-708 | 44.6 |  | hsa-miR-136-3p | 71.8 |
| bta-miR-654 | 103.9 |  | eca-miR-224 | 43.5 |  | bta-miR-146b | 71.8 |
| bta-miR-181d | 102.3 |  | eca-miR-671-3p | 43.5 |  | eca-miR-504 | 71.4 |
| eca-miR-543 | 102.0 |  | hsa-miR-4443 | 42.1 |  | hsa-miR-139-3p | 70.2 |
| eca-miR-129b-3p | 101.5 |  | eca-miR-134 | 40.7 |  | hsa-miR-132-5p | 70.0 |
| hsa-miR-99b-3p | 101.1 |  | hsa-miR-99b-3p | 40.7 |  | eca-miR-155 | 67.9 |
| eca-miR-107b | 98.8 |  | eca-miR-107b | 39.8 |  | bta-miR-488 | 66.2 |
| bta-miR-329b | 97.3 |  | bta-miR-16b | 39.7 |  | eca-miR-1839 | 65.7 |
| hsa-miR-429 | 93.8 |  | eca-miR-374a | 39.7 |  | hsa-miR-219a-2-3p | 65.1 |
| hsa-miR-181a-3p | 93.1 |  | bta-miR-218 | 38.8 |  | eca-miR-214 | 63.6 |
| hsa-miR-382-3p | 91.6 |  | bta-miR-455-5p | 38.4 |  | bta-miR-455-3p | 61.7 |
| eca-miR-142-5p | 89.7 |  | hsa-miR-15b-3p | 37.9 |  | hsa-miR-574-3p | 61.2 |
| bta-miR-199a-5p | 89.1 |  | hsa-miR-200b-5p | 37.9 |  | hsa-miR-708-3p | 61.2 |
| bta-miR-218 | 89.0 |  | eca-miR-1298 | 34.9 |  | bta-miR-129 | 61.1 |
| hsa-miR-155-5p | 88.2 |  | bta-miR-485 | 34.9 |  | hsa-miR-195-3p | 59.3 |
| hsa-miR-145-3p | 87.9 |  | hsa-miR-339-5p | 34.9 |  | bta-miR-218 | 58.9 |
| eca-miR-141 | 84.9 |  | hsa-miR-339-3p | 34.9 |  | bta-miR-654 | 56.9 |
| eca-miR-15b | 84.1 |  | hsa-miR-1843 | 34.4 |  | eca-miR-182 | 56.9 |
| eca-miR-182 | 84.0 |  | hsa-miR-455-5p | 34.4 |  | eca-miR-363 | 56.7 |
| hsa-miR-125b-2-3p | 83.6 |  | eca-miR-32 | 34.4 |  | hsa-miR-501-3p | 56.7 |
| eca-miR-197 | 82.6 |  | bta-miR-329b | 33.5 |  | eca-miR-107b | 55.0 |
| bta-miR-219-3p | 82.4 |  | eca-miR-204b | 33.0 |  | eca-miR-708 | 54.6 |
| eca-miR-340-3p | 78.8 |  | hsa-miR-501-3p | 33.0 |  | eca-miR-758 | 53.3 |
| eca-miR-96 | 76.6 |  | hsa-miR-877-3p | 33.0 |  | eca-miR-15b | 51.1 |
| eca-miR-28-5p | 76.6 |  | hsa-miR-221-5p | 31.6 |  | eca-miR-450a | 51.1 |
| hsa-miR-21-3p | 76.6 |  | hsa-miR-3196 | 31.6 |  | hsa-miR-382-3p | 50.5 |
| bta-miR-16b | 75.1 |  | bta-miR-488 | 31.6 |  | bta-miR-16b | 50.5 |
| bta-miR-378c | 72.9 |  | hsa-miR-16-2-3p | 31.1 |  | eca-miR-485-5p | 50.5 |
| hsa-miR-200b-5p | 71.9 |  | eca-miR-485-3p | 30.7 |  | hsa-miR-130b-5p | 48.8 |
| hsa-miR-320d | 71.0 |  | eca-miR-129a-3p | 25.8 |  | hsa-miR-24-2-5p | 48.3 |
| eca-miR-132 | 69.0 |  | eca-miR-361-5p | 25.8 |  | bta-miR-224 | 48.3 |
| hsa-miR-3158-3p | 64.9 |  | bta-miR-10174-3p | 25.3 |  | hsa-miR-143-5p | 47.0 |
| bta-miR-107 | 63.7 |  | hsa-miR-296-3p | 25.3 |  | hsa-miR-146b-5p | 46.6 |
| bta-miR-488 | 62.8 |  | hsa-miR-1224-5p | 25.3 |  | hsa-miR-3135b | 46.4 |
| hsa-miR-3615 | 61.7 |  | hsa-miR-582-3p | 25.3 |  | bta-miR-329b | 46.4 |
| bta-miR-22-5p | 61.7 |  | bta-miR-150 | 25.3 |  | bta-miR-1307 | 44.9 |
| bta-miR-455-3p | 61.2 |  | hsa-miR-127-5p | 24.4 |  | eca-miR-432 | 44.9 |
| eca-miR-485-3p | 61.1 |  | hsa-miR-92b-5p | 24.4 |  | hsa-miR-668-3p | 44.9 |
| hsa-miR-30c-2-3p | 59.8 |  | eca-miR-383 | 24.4 |  | bta-miR-181b | 44.5 |
| hsa-miR-452-5p | 57.8 |  | hsa-miR-668-3p | 24.4 |  | bta-miR-378c | 44.3 |
| bta-miR-224 | 56.3 |  | hsa-miR-9985 | 24.4 |  | bta-miR-219-3p | 42.5 |
| bta-miR-181b | 56.0 |  | hsa-miR-9-3p | 23.9 |  | eca-miR-676 | 42.1 |
| hsa-miR-221-5p | 55.4 |  | eca-miR-411 | 23.9 |  | eca-miR-196b | 40.4 |
| bta-miR-424-3p | 54.3 |  | bta-miR-126-3p | 23.9 |  | eca-miR-137 | 40.4 |
| eca-miR-409-5p | 53.8 |  | eca-miR-331 | 23.9 |  | bta-miR-142-5p | 40.0 |
| hsa-miR-708-3p | 53.7 |  | eca-miR-590-3p | 23.9 |  | eca-miR-409-5p | 39.5 |
| eca-miR-872 | 51.8 |  | hsa-miR-769-5p | 23.9 |  | bta-miR-424-3p | 38.2 |
| hsa-miR-589-5p | 51.3 |  | eca-miR-338-5p | 23.5 |  | hsa-miR-155-5p | 35.9 |
| hsa-miR-139-3p | 49.9 |  | hsa-miR-584-5p | 23.5 |  | eca-miR-361-5p | 35.9 |
| bta-miR-485 | 49.8 |  | hsa-miR-1180-3p | 23.5 |  | hsa-miR-214-5p | 34.2 |
| bta-let-7a-3p | 48.3 |  | hsa-miR-146b-3p | 22.5 |  | bta-miR-17-5p | 34.2 |
| hsa-miR-15b-3p | 44.8 |  | bta-miR-2881 | 22.5 |  | eca-miR-495 | 34.2 |
| hsa-miR-584-5p | 44.7 |  | hsa-miR-1301-3p | 22.5 |  | hsa-miR-27b-5p | 34.2 |
| eca-miR-140-5p | 43.1 |  | hsa-miR-132-5p | 22.1 |  | eca-miR-224 | 34.2 |
| bta-miR-181a | 43.0 |  | eca-miR-454 | 22.1 |  | hsa-miR-4443 | 34.2 |
| bta-miR-2428 | 42.7 |  | bta-miR-1839 | 16.7 |  | hsa-miR-769-5p | 33.7 |
| eca-miR-135b | 42.5 |  | bta-miR-107 | 16.7 |  | hsa-let-7b-3p | 32.0 |
| eca-miR-196b | 41.3 |  | hsa-miR-135a-2-3p | 16.7 |  | bta-miR-503-5p | 32.0 |
| bta-miR-183 | 41.0 |  | hsa-miR-4433b-5p | 16.7 |  | hsa-miR-320b | 31.6 |
| eca-miR-542-3p | 41.0 |  | hsa-let-7f-2-3p | 16.7 |  | hsa-miR-654-5p | 31.6 |
| eca-miR-374a | 41.0 |  | bta-miR-452 | 16.7 |  | eca-miR-34a | 29.7 |
| hsa-miR-501-3p | 40.8 |  | hsa-miR-651-5p | 16.7 |  | eca-miR-342-3p | 29.7 |
| eca-miR-485-5p | 40.8 |  | bta-miR-411a | 16.3 |  | hsa-miR-1268a | 29.7 |
| hsa-miR-431-3p | 39.2 |  | hsa-miR-210-3p | 16.3 |  | hsa-let-7a-2-3p | 29.2 |
| eca-miR-151-5p | 37.5 |  | hsa-miR-548k | 16.3 |  | eca-miR-28-5p | 27.5 |
| hsa-miR-548o-3p | 37.3 |  | eca-miR-652 | 16.3 |  | eca-miR-369-3p | 27.5 |
| hsa-miR-1268a | 37.2 |  | hsa-miR-4455 | 16.3 |  | hsa-miR-548o-3p | 27.5 |
| hsa-miR-7706 | 37.1 |  | eca-miR-409-5p | 16.3 |  | bta-miR-1224 | 27.5 |
| eca-miR-671-3p | 37.0 |  | hsa-miR-708-3p | 16.3 |  | eca-miR-193a-5p | 27.1 |
| eca-miR-106b | 35.6 |  | bta-miR-323 | 16.3 |  | eca-miR-1271a | 27.1 |
| eca-miR-185 | 35.5 |  | eca-miR-365 | 16.3 |  | eca-miR-374a | 27.1 |
| hsa-miR-769-5p | 35.5 |  | eca-miR-135b | 16.3 |  | eca-miR-125a-3p | 25.3 |
| hsa-miR-143-5p | 35.4 |  | eca-miR-139-5p | 16.3 |  | hsa-miR-30c-2-3p | 25.3 |
| hsa-miR-425-5p | 35.3 |  | eca-miR-744 | 16.3 |  | bta-miR-323 | 25.3 |
| eca-miR-450a | 35.3 |  | eca-miR-19b | 16.3 |  | eca-miR-138 | 23.0 |
| hsa-miR-516b-5p | 33.6 |  | hsa-miR-7977 | 16.3 |  | bta-miR-22-5p | 23.0 |
| eca-miR-129a-3p | 31.9 |  | eca-miR-30e | 14.9 |  | hsa-miR-3196 | 23.0 |
| hsa-miR-132-5p | 31.7 |  | eca-miR-323-3p | 14.9 |  | bta-miR-11975 | 23.0 |
| eca-miR-502-3p | 30.0 |  | eca-miR-504 | 14.9 |  | eca-miR-223 | 21.3 |
| eca-miR-139-5p | 30.0 |  | hsa-miR-26b-3p | 14.9 |  | hsa-miR-148a-5p | 21.3 |
| hsa-miR-1180-3p | 30.0 |  | hsa-miR-675-3p | 14.9 |  | eca-miR-338-5p | 21.3 |
| hsa-miR-181c-3p | 29.7 |  | eca-miR-139-3p | 14.9 |  | hsa-miR-425-5p | 21.3 |
| eca-miR-708 | 29.7 |  | hsa-miR-6842-3p | 14.9 |  | hsa-miR-369-5p | 21.3 |
| hsa-miR-27b-5p | 29.7 |  |  |  |  | eca-miR-331 | 21.3 |
| eca-miR-365 | 29.7 |  |  |  |  | hsa-miR-582-3p | 21.3 |
| bta-miR-339a | 29.4 |  |  |  |  | eca-miR-29b | 21.3 |
| bta-miR-34c | 28.1 |  |  |  |  | eca-miR-135b | 21.3 |
| eca-miR-660 | 28.1 |  |  |  |  | eca-miR-494 | 21.3 |
| eca-miR-187 | 28.1 |  |  |  |  | eca-miR-140-5p | 20.8 |
| eca-miR-432 | 27.9 |  |  |  |  | bta-miR-212 | 20.8 |
| eca-miR-504 | 27.8 |  |  |  |  | eca-miR-139-5p | 20.8 |
| hsa-miR-16-2-3p | 26.2 |  |  |  |  | eca-miR-126-5p | 19.1 |
| hsa-miR-455-5p | 26.1 |  |  |  |  | eca-miR-31 | 19.1 |
| eca-miR-338-5p | 26.0 |  |  |  |  | eca-miR-193b | 19.1 |
| eca-miR-32 | 25.9 |  |  |  |  | bta-miR-485 | 19.1 |
| eca-miR-495 | 24.3 |  |  |  |  | bta-miR-107 | 19.1 |
| bta-miR-760-3p | 24.2 |  |  |  |  | hsa-miR-1260a | 19.1 |
| eca-miR-335 | 24.2 |  |  |  |  | hsa-miR-500a-3p | 19.1 |
| hsa-miR-339-3p | 24.2 |  |  |  |  | hsa-miR-431-3p | 19.1 |
| eca-miR-29b | 22.4 |  |  |  |  | hsa-let-7e-3p | 19.1 |
| hsa-miR-369-5p | 22.3 |  |  |  |  | hsa-miR-30d-3p | 19.1 |
| hsa-miR-500a-3p | 22.3 |  |  |  |  | hsa-miR-6131 | 19.1 |
| bta-miR-323 | 22.3 |  |  |  |  | hsa-miR-1224-5p | 19.1 |
| hsa-miR-7974 | 22.1 |  |  |  |  | bta-miR-199c | 19.1 |
| hsa-miR-128-1-5p | 22.1 |  |  |  |  | hsa-miR-589-5p | 19.1 |
| eca-miR-454 | 22.1 |  |  |  |  | eca-miR-744 | 19.1 |
| hsa-miR-214-5p | 22.0 |  |  |  |  | eca-miR-346 | 19.1 |
| hsa-miR-374a-3p | 22.0 |  |  |  |  | eca-miR-19b | 19.1 |
| hsa-miR-340-3p | 20.6 |  |  |  |  | eca-miR-872 | 14.6 |
| bta-miR-150 | 20.6 |  |  |  |  | eca-miR-362-5p | 14.6 |
| hsa-miR-629-5p | 20.6 |  |  |  |  | hsa-miR-181a-3p | 14.6 |
| eca-miR-153 | 20.6 |  |  |  |  | eca-miR-383 | 14.6 |
| hsa-miR-325 | 20.4 |  |  |  |  | eca-miR-507a | 14.6 |
| eca-miR-29c | 20.4 |  |  |  |  | eca-miR-532-3p | 14.6 |
| hsa-miR-503-5p | 20.4 |  |  |  |  | hsa-miR-411-3p | 14.6 |
| hsa-miR-185-3p | 18.7 |  |  |  |  | eca-miR-32 | 14.6 |
| hsa-miR-576-3p | 18.5 |  |  |  |  |  |  |
| bta-miR-212 | 18.5 |  |  |  |  |  |  |
| eca-miR-8986a | 18.5 |  |  |  |  |  |  |
| hsa-miR-378c | 16.8 |  |  |  |  |  |  |
| bta-miR-93 | 16.8 |  |  |  |  |  |  |
| hsa-miR-1271-5p | 16.8 |  |  |  |  |  |  |
| hsa-miR-625-3p | 16.8 |  |  |  |  |  |  |
| bta-miR-421 | 16.6 |  |  |  |  |  |  |
| hsa-miR-668-3p | 16.6 |  |  |  |  |  |  |
| hsa-miR-1303 | 16.6 |  |  |  |  |  |  |
| hsa-miR-766-3p | 14.9 |  |  |  |  |  |  |
| bta-miR-12034 | 14.9 |  |  |  |  |  |  |
| bta-miR-2881 | 14.9 |  |  |  |  |  |  |
| hsa-miR-582-3p | 14.9 |  |  |  |  |  |  |
| hsa-miR-509-3-5p | 14.9 |  |  |  |  |  |  |
| eca-miR-383 | 14.8 |  |  |  |  |  |  |
| eca-miR-138 | 14.8 |  |  |  |  |  |  |
| eca-miR-130a | 14.8 |  |  |  |  |  |  |
| hsa-miR-30d-3p | 12.9 |  |  |  |  |  |  |
| bta-miR-1224 | 12.9 |  |  |  |  |  |  |
| bta-miR-196b | 12.9 |  |  |  |  |  |  |
| hsa-miR-1304-3p | 12.9 |  |  |  |  |  |  |

| **Table S3: Differentially expressed (DE) miRNAs in the extracellular vesicles obtained from follicular fluids of the different follicular stages** | | | | | |
| --- | --- | --- | --- | --- | --- |
|  |  |  |  |  |  |
| **G *vs.* D** |  |  |  |  |  |
| **Name** | **Feature ID** | **miRBase accession** | **Fold Change** | **P-value** | **FDR** |
| hsa-miR-3135b | GGCTGGAGCGAGTGCAGTGGTG | [MI0016809](https://www.mirbase.org/cgi-bin/mirna_entry.pl?acc=MI0016809) | 288.64 | 0.002328 | 0.052692 |
| bta-miR-11980 | AGGCAACGGGCTTGGCGGAG | [MI0038192](https://www.mirbase.org/cgi-bin/mirna_entry.pl?acc=MI0038192) | 36.77 | 1.63E-05 | 0.000837 |
| eca-miR-126-5p | CATTATTACTTTTGGTACGCG | [MI0000471](https://www.mirbase.org/cgi-bin/mirna_entry.pl?acc=MI0000471) | 29.83 | 0.006563 | 0.093711 |
| hsa-miR-9-3p | ATAAAGCTAGATAACCGAAAGT | [MI0000466](https://www.mirbase.org/cgi-bin/mirna_entry.pl?acc=MI0000466) | 18.44 | 0.003523 | 0.06468 |
| bta-miR-9-5p | TCTTTGGTTATCTAGCTGTATG | [MI0009912](https://www.mirbase.org/cgi-bin/mirna_entry.pl?acc=MI0009912) | 16.62 | 1.69E-06 | 0.000122 |
| eca-miR-184 | TGGACGGAGAACTGATAAGGGT | [MI0000481](https://www.mirbase.org/cgi-bin/mirna_entry.pl?acc=MI0000481) | 15.13 | 9.64E-05 | 0.004131 |
| eca-miR-9a | TCTTTGGTTATCTAGCTGTATGA | [MI0000466](https://www.mirbase.org/cgi-bin/mirna_entry.pl?acc=MI0000466) | 14.26 | 8.7E-07 | 0.000112 |
| bta-miR-2478 | GTATCCCACTTCTGACACCA | [MI0011540](https://www.mirbase.org/cgi-bin/mirna_entry.pl?acc=MI0011540) | 13.96 | 0.000394 | 0.014453 |
| eca-miR-615-3p | TCCGAGCCTGGGTCTCCCTCTC | [MI0012729](https://www.mirbase.org/cgi-bin/mirna_entry.pl?acc=MI0012729) | 13.74 | 0.00246 | 0.052692 |
| eca-miR-379 | TGGTAGACTATGGAACGTAGG | [MI0000787](https://www.mirbase.org/cgi-bin/mirna_entry.pl?acc=MI0000787) | 11.95 | 0.00645 | 0.093711 |
| bta-miR-200b | TAATACTGCCTGGTAATGATG | [MI0005055](https://www.mirbase.org/cgi-bin/mirna_entry.pl?acc=MI0005055) | 11.71 | 0.002678 | 0.052934 |
| eca-miR-124 | TAAGGCACGCGGTGAATGCC | [MI0012662](https://www.mirbase.org/cgi-bin/mirna_entry.pl?acc=MI0012662) | 8.67 | 0.007068 | 0.095603 |
| eca-miR-381 | TATACAAGGGCAAGCTCTCTGT | [MI0000789](https://www.mirbase.org/cgi-bin/mirna_entry.pl?acc=MI0000789) | 7.44 | 0.005613 | 0.093711 |
| bta-miR-215 | ATGACCTATGAATTGACAGACA | [MI0005016](https://www.mirbase.org/cgi-bin/mirna_entry.pl?acc=MI0005016) | -4.73 | 0.001201 | 0.030868 |
| eca-miR-148a | TCAGTGCACTACAGAACTTTGT | [MI0000253](https://www.mirbase.org/cgi-bin/mirna_entry.pl?acc=MI0000253) | -10.16 | 0.000739 | 0.021095 |
| hsa-miR-4492 | GGGGCTGGGCGCGCGCC | [MI0016854](https://www.mirbase.org/cgi-bin/mirna_entry.pl?acc=MI0016854) | -10.90 | 1.91E-06 | 0.000122 |
| hsa-miR-155-5p | TTAATGCTAATCGTGATAGGGGTT | [MI0000681](https://www.mirbase.org/cgi-bin/mirna_entry.pl?acc=MI0000681) | -12.68 | 0.006148 | 0.093711 |
| hsa-miR-4497 | CTCCGGGACGGCTGGGC | [MI0016859](https://www.mirbase.org/cgi-bin/mirna_entry.pl?acc=MI0016859) | -14.64 | 4.07E-09 | 1.05E-06 |
| eca-miR-378 | ACTGGACTTGGAGTCAGAAGG | [MI0012812](https://www.mirbase.org/cgi-bin/mirna_entry.pl?acc=MI0012812) | -53.26 | 0.000584 | 0.018752 |
|  |  |  |  |  |  |
|  |  |  |  |  |  |
| **P *vs.* D** |  |  |  |  |  |
| **Name** | **Feature ID** | **miRBase accession** | **Fold Change** | **P-value** | **FDR** |
| bta-miR-199a-5p | CCCAGTGTTCAGACTACCTGTT | [MI0004758](https://www.mirbase.org/cgi-bin/mirna_entry.pl?acc=MI0004758) | 169.52 | 0.003977 | 0.054555 |
| bta-miR-574 | TGAGTGTGTGTGTGTGAGTGTGTG | [MI0021122](https://www.mirbase.org/cgi-bin/mirna_entry.pl?acc=MI0021122) | 131.55 | 0.012291 | 0.098642 |
| hsa-miR-493-5p | TTGTACATGGTAGGCTTTCATT | [MI0003132](https://www.mirbase.org/cgi-bin/mirna_entry.pl?acc=MI0003132) | 87.47 | 0.010373 | 0.097482 |
| eca-miR-133a | TTTGGTCCCCTTCAACCAGCTG | [MI0000450](https://www.mirbase.org/cgi-bin/mirna_entry.pl?acc=MI0000450) | 78.03 | 0.012593 | 0.098642 |
| hsa-miR-576-3p | AAGATGTGGAAAAATTGGAATC | [MI0003583](https://www.mirbase.org/cgi-bin/mirna_entry.pl?acc=MI0003583) | 57.47 | 0.00439 | 0.054555 |
| bta-miR-455-5p | TATGTGCCTTTGGACTACATC | [MI0005049](https://www.mirbase.org/cgi-bin/mirna_entry.pl?acc=MI0005049) | 28.89 | 0.000834 | 0.018421 |
| eca-miR-184 | TGGACGGAGAACTGATAAGGGT | [MI0000481](https://www.mirbase.org/cgi-bin/mirna_entry.pl?acc=MI0000481) | 21.66 | 1.01E-05 | 0.000789 |
| eca-miR-199b-5p | CCCAGTGTTTAGACTATCTGTTC | [MI0000282](https://www.mirbase.org/cgi-bin/mirna_entry.pl?acc=MI0000282) | 16.07 | 0.002471 | 0.038708 |
| bta-miR-9-5p | TCTTTGGTTATCTAGCTGTATG | [MI0009912](https://www.mirbase.org/cgi-bin/mirna_entry.pl?acc=MI0009912) | 14.76 | 4.61E-06 | 0.00068 |
| eca-miR-615-3p | TCCGAGCCTGGGTCTCCCTCTC | [MI0012729](https://www.mirbase.org/cgi-bin/mirna_entry.pl?acc=MI0012729) | 11.43 | 0.004971 | 0.054555 |
| eca-miR-9a | TCTTTGGTTATCTAGCTGTATGA | [MI0000466](https://www.mirbase.org/cgi-bin/mirna_entry.pl?acc=MI0000466) | 9.07 | 4.56E-05 | 0.002142 |
| eca-miR-381 | TATACAAGGGCAAGCTCTCTGT | [MI0000789](https://www.mirbase.org/cgi-bin/mirna_entry.pl?acc=MI0000789) | 8.62 | 0.002969 | 0.043601 |
| eca-miR-423-3p | AGCTCGGTCTGAGGCCCCTCAGT | [MI0001445](https://www.mirbase.org/cgi-bin/mirna_entry.pl?acc=MI0001445) | 4.26 | 0.008592 | 0.084127 |
| eca-miR-92a | TATTGCACTTGTCCCGGCCTGT | [MI0000093](https://www.mirbase.org/cgi-bin/mirna_entry.pl?acc=MI0000093) | -2.83 | 0.01172 | 0.098362 |
| eca-miR-183 | TATGGCACTGGTAGAATTCACT | [MI0000273](https://www.mirbase.org/cgi-bin/mirna_entry.pl?acc=MI0000273) | -7.21 | 0.0081 | 0.082763 |
| eca-miR-486-5p | TCCTGTACTGAGCTGCCCCGAG | [MI0002470](https://www.mirbase.org/cgi-bin/mirna_entry.pl?acc=MI0002470) | -7.26 | 8.11E-05 | 0.002723 |
| eca-miR-20a | TAAAGTGCTTATAGTGCAGGTAG | [MI0000076](https://www.mirbase.org/cgi-bin/mirna_entry.pl?acc=MI0000076) | -7.28 | 0.010785 | 0.097482 |
| eca-miR-93 | CAAAGTGCTGTTCGTGCAGGTAG | [MI0000095](https://www.mirbase.org/cgi-bin/mirna_entry.pl?acc=MI0000095) | -7.91 | 0.011316 | 0.098362 |
| eca-miR-148a | TCAGTGCACTACAGAACTTTGT | [MI0000253](https://www.mirbase.org/cgi-bin/mirna_entry.pl?acc=MI0000253) | -9.20 | 0.001237 | 0.021033 |
| eca-miR-23a | ATCACATTGCCAGGGATTTCC | [MI0000079](https://www.mirbase.org/cgi-bin/mirna_entry.pl?acc=MI0000079) | -11.05 | 0.005107 | 0.054555 |
| bta-miR-27a-3p | TTCACAGTGGCTAAGTTCCG | [MI0004746](https://www.mirbase.org/cgi-bin/mirna_entry.pl?acc=MI0004746) | -11.51 | 0.000863 | 0.018421 |
| eca-miR-155 | TTAATGCTAATCGTGATAGGGGT | [MI0009752](https://www.mirbase.org/cgi-bin/mirna_entry.pl?acc=MI0009752) | -16.44 | 0.001253 | 0.021033 |
| hsa-miR-155-5p | TTAATGCTAATCGTGATAGGGGTT | [MI0000681](https://www.mirbase.org/cgi-bin/mirna_entry.pl?acc=MI0000681) | -33.17 | 0.00055 | 0.014364 |
| bta-miR-142-5p | CATAAAGTAGAAAGCACTAC | [MI0005011](https://www.mirbase.org/cgi-bin/mirna_entry.pl?acc=MI0005011) | -38.27 | 1.34E-05 | 0.000789 |
| eca-miR-146b-5p | TGAGAACTGAATTCCATAGGCT | [MI0012648](https://www.mirbase.org/cgi-bin/mirna_entry.pl?acc=MI0012648) | -55.24 | 0.000228 | 0.006696 |
| eca-miR-31 | AGGCAAGATGCTGGCATAGCT | [MI0000089](https://www.mirbase.org/cgi-bin/mirna_entry.pl?acc=MI0000089) | -80.70 | 6.73E-05 | 0.002634 |
| eca-miR-200a | TAACACTGTCTGGTAACGATGT | [MI0000737](https://www.mirbase.org/cgi-bin/mirna_entry.pl?acc=MI0000737) | -85.51 | 5.78E-06 | 0.00068 |
| eca-miR-205 | TCCTTCATTCCACCGGAGTCTG | [MI0000285](https://www.mirbase.org/cgi-bin/mirna_entry.pl?acc=MI0000285) | -985.09 | 0.004867 | 0.054555 |
|  |  |  |  |  |  |
|  |  |  |  |  |  |
| **P *vs.* G** |  |  |  |  |  |
| **Name** | **Feature ID** | **miRBase accession** | **Fold Change** | **P-value** | **FDR** |
| eca-miR-215 | ATGACCTATGAATTGACAGAC | [MI0000291](https://www.mirbase.org/cgi-bin/mirna_entry.pl?acc=MI0000291) | 15.68 | 0.00196 | 0.065517 |
| hsa-miR-4497 | CTCCGGGACGGCTGGGC | [MI0016859](https://www.mirbase.org/cgi-bin/mirna_entry.pl?acc=MI0016859) | 6.95 | 2.27E-05 | 0.003099 |
| bta-miR-215 | ATGACCTATGAATTGACAGACA | [MI0005016](https://www.mirbase.org/cgi-bin/mirna_entry.pl?acc=MI0005016) | 4.24 | 0.00264 | 0.065517 |
| hsa-miR-4492 | GGGGCTGGGCGCGCGCC | [MI0016854](https://www.mirbase.org/cgi-bin/mirna_entry.pl?acc=MI0016854) | 4.24 | 0.004161 | 0.081133 |
| eca-miR-486-5p | TCCTGTACTGAGCTGCCCCGAG | [MI0002470](https://www.mirbase.org/cgi-bin/mirna_entry.pl?acc=MI0002470) | -3.90 | 0.006893 | 0.098589 |
| bta-miR-2478 | GTATCCCACTTCTGACACCA | [MI0011540](https://www.mirbase.org/cgi-bin/mirna_entry.pl?acc=MI0011540) | -8.07 | 0.004971 | 0.084818 |
| bta-miR-142-5p | CATAAAGTAGAAAGCACTAC | [MI0005011](https://www.mirbase.org/cgi-bin/mirna_entry.pl?acc=MI0005011) | -12.88 | 0.002411 | 0.065517 |
| hsa-miR-4488 | AGGGGGCGGGCTCCGGCG | [MI0016849](https://www.mirbase.org/cgi-bin/mirna_entry.pl?acc=MI0016849) | -12.93 | 0.003943 | 0.081133 |
| eca-miR-200c | TAATACTGCCGGGTAATGATGGA | [MI0000650](https://www.mirbase.org/cgi-bin/mirna_entry.pl?acc=MI0000650) | -20.55 | 0.001712 | 0.065517 |
| eca-miR-146b-5p | TGAGAACTGAATTCCATAGGCT | [MI0012648](https://www.mirbase.org/cgi-bin/mirna_entry.pl?acc=MI0012648) | -23.53 | 0.003822 | 0.081133 |
| hsa-miR-146b-5p | TGAGAACTGAATTCCATAGGCTG | [MI0003129](https://www.mirbase.org/cgi-bin/mirna_entry.pl?acc=MI0003129) | -30.27 | 5.17E-05 | 0.003754 |
| bta-miR-11980 | AGGCAACGGGCTTGGCGGAG | [MI0038192](https://www.mirbase.org/cgi-bin/mirna_entry.pl?acc=MI0038192) | -36.30 | 1.75E-05 | 0.003099 |
| eca-miR-31 | AGGCAAGATGCTGGCATAGCT | [MI0000089](https://www.mirbase.org/cgi-bin/mirna_entry.pl?acc=MI0000089) | -49.05 | 0.000416 | 0.018917 |
| eca-miR-200a | TAACACTGTCTGGTAACGATGT | [MI0000737](https://www.mirbase.org/cgi-bin/mirna_entry.pl?acc=MI0000737) | -52.50 | 5.5E-05 | 0.003754 |
| eca-miR-205 | TCCTTCATTCCACCGGAGTCTG | [MI0000285](https://www.mirbase.org/cgi-bin/mirna_entry.pl?acc=MI0000285) | -718.32 | 0.007223 | 0.098589 |

| **Table S4: Clusters of miRNAs with different expression patterns among the different follicular stages** | | |
| --- | --- | --- |
| **miRNA** | **CLUSTER** | **MEM.SHIP** |
| eca-miR-193b | 1 | 0.8315716 |
| eca-miR-507a | 1 | 0.8315716 |
| eca-miR-99a | 1 | 0.8109369 |
| hsa-let-7d-3p | 1 | 0.6966116 |
| hsa-miR-576-3p | 1 | 0.6797606 |
| hsa-miR-7704 | 1 | 0.6585379 |
| hsa-miR-378d | 1 | 0.5878902 |
| hsa-miR-92b-5p | 1 | 0.5642134 |
| eca-miR-671-5p | 1 | 0.5620448 |
| hsa-miR-514a-3p | 1 | 0.5436897 |
| eca-miR-222 | 1 | 0.5420402 |
| hsa-miR-130b-5p | 1 | 0.5414778 |
| hsa-miR-516b-5p | 1 | 0.5374842 |
| eca-miR-671-3p | 1 | 0.5229054 |
| bta-miR-99a-5p | 1 | 0.5214834 |
| hsa-miR-200a-5p | 1 | 0.5201627 |
| bta-let-7a-3p | 1 | 0.5105257 |
| bta-let-7b | 1 | 0.47074 |
| eca-miR-125a-3p | 1 | 0.4346459 |
| eca-miR-206 | 1 | 0.4252765 |
| hsa-miR-574-5p | 1 | 0.399915 |
| eca-miR-7 | 1 | 0.3966526 |
| bta-miR-2881 | 1 | 0.391905 |
| bta-miR-199a-5p | 1 | 0.3221077 |
| bta-miR-182 | 1 | 0.3213249 |
| eca-miR-196a | 1 | 0.3198081 |
| bta-miR-1224 | 1 | 0.3153723 |
| eca-miR-143 | 1 | 0.3121736 |
| hsa-miR-200b-5p | 1 | 0.2942434 |
| hsa-miR-139-3p | 1 | 0.2896907 |
| hsa-let-7b-3p | 1 | 0.2704379 |
| hsa-miR-132-5p | 1 | 0.2699459 |
| eca-miR-10b | 1 | 0.2608544 |
| hsa-miR-125b-2-3p | 1 | 0.2547933 |
| hsa-let-7e-3p | 1 | 0.2526951 |
| eca-miR-504 | 1 | 0.2478542 |
| hsa-miR-668-3p | 1 | 0.2411192 |
| bta-miR-493 | 1 | 0.2388711 |
| bta-miR-221 | 1 | 0.2383909 |
| eca-miR-758 | 1 | 0.232809 |
| hsa-let-7a-2-3p | 1 | 0.2230073 |
| bta-miR-488 | 2 | 0.7427566 |
| eca-miR-365 | 2 | 0.7421448 |
| hsa-miR-654-5p | 2 | 0.7084373 |
| eca-miR-323-3p | 2 | 0.5702369 |
| hsa-miR-210-3p | 2 | 0.5547517 |
| eca-miR-133a | 2 | 0.5348602 |
| bta-miR-181d | 2 | 0.453046 |
| eca-miR-494 | 2 | 0.4415115 |
| hsa-miR-708-3p | 2 | 0.4291984 |
| bta-miR-212 | 2 | 0.4205813 |
| eca-miR-127 | 2 | 0.4157562 |
| eca-miR-381 | 2 | 0.3929201 |
| eca-miR-196b | 2 | 0.3630403 |
| hsa-miR-99b-3p | 2 | 0.3403232 |
| eca-let-7c | 2 | 0.3316335 |
| hsa-miR-27b-5p | 2 | 0.3251055 |
| hsa-miR-369-5p | 2 | 0.3132774 |
| bta-miR-10174-3p | 2 | 0.3074462 |
| eca-miR-181b | 2 | 0.3054083 |
| eca-miR-29b | 2 | 0.3040912 |
| eca-miR-370 | 2 | 0.3009185 |
| hsa-miR-136-3p | 2 | 0.294318 |
| eca-miR-423-3p | 2 | 0.2927317 |
| eca-let-7e | 2 | 0.2901401 |
| bta-miR-143 | 2 | 0.2896564 |
| bta-miR-9-5p | 2 | 0.2771541 |
| hsa-miR-500a-3p | 2 | 0.2599382 |
| hsa-miR-6131 | 2 | 0.2590071 |
| hsa-miR-769-5p | 2 | 0.2541537 |
| eca-miR-543 | 2 | 0.2453362 |
| eca-miR-184 | 2 | 0.2401638 |
| eca-miR-346 | 2 | 0.2384529 |
| eca-miR-615-3p | 2 | 0.2342896 |
| hsa-miR-30d-3p | 2 | 0.2319119 |
| hsa-miR-26b-5p | 2 | 0.2096172 |
| eca-miR-1271a | 2 | 0.203935 |
| bta-miR-200a | 2 | 0.2037659 |
| hsa-miR-548o-3p | 2 | 0.2013127 |
| eca-miR-204b | 2 | 0.200261 |
| hsa-miR-1268a | 2 | 0.1990778 |
| hsa-miR-155-5p | 3 | 0.834294 |
| eca-miR-451 | 3 | 0.8317694 |
| eca-miR-192 | 3 | 0.6899944 |
| eca-miR-183 | 3 | 0.6562895 |
| eca-miR-98 | 3 | 0.6469901 |
| eca-miR-383 | 3 | 0.6288301 |
| hsa-miR-9985 | 3 | 0.6147163 |
| hsa-miR-452-5p | 3 | 0.6144713 |
| eca-miR-590-3p | 3 | 0.6078753 |
| hsa-miR-4508 | 3 | 0.602573 |
| bta-miR-424-3p | 3 | 0.5993206 |
| hsa-miR-548k | 3 | 0.5751333 |
| bta-miR-455-3p | 3 | 0.5535452 |
| eca-miR-148a | 3 | 0.5394888 |
| hsa-miR-4433b-5p | 3 | 0.4918604 |
| hsa-miR-651-5p | 3 | 0.4918604 |
| hsa-miR-214-5p | 3 | 0.4661934 |
| eca-miR-140-5p | 3 | 0.4609929 |
| eca-miR-148b-3p | 3 | 0.4601026 |
| hsa-miR-26b-3p | 3 | 0.4589087 |
| bta-miR-185 | 3 | 0.4486037 |
| eca-miR-22 | 3 | 0.4476583 |
| hsa-miR-3615 | 3 | 0.4278233 |
| eca-miR-150 | 3 | 0.4274705 |
| eca-miR-486-3p | 3 | 0.4107338 |
| hsa-miR-143-5p | 3 | 0.4089245 |
| eca-miR-433 | 3 | 0.3990619 |
| hsa-miR-877-3p | 3 | 0.3844688 |
| eca-miR-1839 | 3 | 0.3767967 |
| hsa-miR-1290 | 3 | 0.3675852 |
| bta-miR-11976 | 3 | 0.3666237 |
| hsa-miR-361-3p | 3 | 0.3604856 |
| bta-miR-378 | 3 | 0.3498052 |
| bta-miR-11975 | 3 | 0.3386435 |
| eca-miR-27a | 3 | 0.3356924 |
| eca-miR-122 | 3 | 0.3325162 |
| hsa-miR-146b-3p | 3 | 0.3209471 |
| bta-miR-1839 | 3 | 0.320596 |
| hsa-miR-223-5p | 3 | 0.3106049 |
| hsa-miR-941 | 3 | 0.3094111 |
| eca-miR-93 | 3 | 0.3059415 |
| hsa-miR-675-5p | 3 | 0.3015632 |
| eca-miR-450a | 3 | 0.2942249 |
| eca-miR-378 | 3 | 0.2846144 |
| bta-miR-183 | 3 | 0.2718567 |
| hsa-miR-1301-3p | 3 | 0.2638663 |
| hsa-miR-296-3p | 3 | 0.2631162 |
| eca-miR-146a | 3 | 0.2617758 |
| bta-miR-760-3p | 3 | 0.253255 |
| hsa-miR-4455 | 3 | 0.2532146 |
| eca-miR-506a | 3 | 0.2509044 |
| hsa-miR-4492 | 3 | 0.2460388 |
| hsa-miR-3195 | 3 | 0.2451742 |
| bta-miR-142-5p | 3 | 0.2430945 |
| hsa-let-7f-2-3p | 3 | 0.2376064 |
| bta-miR-452 | 3 | 0.2376064 |
| hsa-miR-629-5p | 3 | 0.2366411 |
| hsa-miR-320d | 3 | 0.2361159 |
| bta-miR-744 | 3 | 0.2316999 |
| hsa-miR-125b-1-3p | 3 | 0.2268442 |
| bta-miR-1246 | 3 | 0.2253435 |
| hsa-miR-4497 | 3 | 0.2114947 |
| hsa-miR-10400-5p | 3 | 0.2053503 |
| eca-miR-17 | 3 | 0.2019836 |
| bta-miR-320a | 3 | 0.1876918 |
| hsa-miR-582-3p | 3 | 0.1730285 |
| eca-miR-532-5p | 3 | 0.1691288 |
| eca-miR-499-5p | 4 | 0.8335965 |
| hsa-miR-1303 | 4 | 0.8255878 |
| eca-miR-338-5p | 4 | 0.8163091 |
| bta-miR-181b | 4 | 0.7743711 |
| eca-miR-101 | 4 | 0.7433397 |
| hsa-miR-3168 | 4 | 0.6957066 |
| eca-miR-221 | 4 | 0.6753667 |
| hsa-miR-30a-3p | 4 | 0.636038 |
| bta-miR-425-5p | 4 | 0.6270943 |
| hsa-miR-195-3p | 4 | 0.5890675 |
| eca-miR-29a | 4 | 0.5615399 |
| eca-miR-200c | 4 | 0.5144382 |
| eca-miR-485-3p | 4 | 0.5132692 |
| eca-miR-23b | 4 | 0.4966415 |
| bta-miR-485 | 4 | 0.4749735 |
| eca-miR-28-3p | 4 | 0.4281397 |
| hsa-miR-24-2-5p | 4 | 0.4204093 |
| hsa-miR-625-3p | 4 | 0.4102413 |
| hsa-miR-185-3p | 4 | 0.3984795 |
| eca-miR-129b-3p | 4 | 0.3648114 |
| eca-miR-328 | 4 | 0.3628348 |
| eca-miR-223 | 4 | 0.3353547 |
| hsa-miR-584-5p | 4 | 0.312585 |
| eca-miR-340-5p | 4 | 0.3079087 |
| eca-miR-363 | 4 | 0.2987424 |
| hsa-miR-30e-3p | 4 | 0.29712 |
| eca-miR-27b | 4 | 0.2899663 |
| eca-miR-145 | 4 | 0.2896854 |
| bta-miR-330 | 4 | 0.2890932 |
| eca-miR-502-3p | 4 | 0.2792345 |
| hsa-miR-6842-3p | 4 | 0.2777502 |
| hsa-miR-429 | 4 | 0.2756589 |
| bta-miR-30a-5p | 4 | 0.2746378 |
| bta-let-7i | 4 | 0.268906 |
| eca-miR-129a-3p | 4 | 0.2646656 |
| eca-let-7g | 4 | 0.2635171 |
| bta-miR-1307 | 4 | 0.2621173 |
| eca-miR-200b | 4 | 0.2616573 |
| eca-miR-28-5p | 4 | 0.250771 |
| hsa-miR-181a-3p | 4 | 0.248982 |
| eca-miR-140-3p | 4 | 0.2466631 |
| bta-miR-23a | 4 | 0.2435205 |
| eca-miR-107a | 4 | 0.2419821 |
| eca-miR-191a | 4 | 0.2266502 |
| hsa-miR-15b-3p | 4 | 0.2110672 |
| bta-miR-93 | 4 | 0.1979671 |
| eca-miR-342-3p | 4 | 0.1889045 |
| bta-miR-11980 | 5 | 0.8549674 |
| eca-miR-30c | 5 | 0.8492191 |
| hsa-miR-766-3p | 5 | 0.847885 |
| hsa-miR-378c | 5 | 0.847885 |
| bta-miR-34c | 5 | 0.847885 |
| hsa-miR-7974 | 5 | 0.847885 |
| eca-miR-130a | 5 | 0.847885 |
| bta-miR-12034 | 5 | 0.847885 |
| hsa-miR-509-3-5p | 5 | 0.847885 |
| eca-miR-8986a | 5 | 0.847885 |
| eca-miR-151-5p | 5 | 0.847885 |
| hsa-miR-1304-3p | 5 | 0.847885 |
| eca-miR-153 | 5 | 0.847885 |
| eca-miR-744 | 5 | 0.758461 |
| eca-miR-1298 | 5 | 0.6567598 |
| eca-miR-126-5p | 5 | 0.6036608 |
| hsa-miR-219a-2-3p | 5 | 0.5958393 |
| hsa-miR-1260a | 5 | 0.5660936 |
| hsa-miR-4443 | 5 | 0.5534955 |
| hsa-miR-345-5p | 5 | 0.5474628 |
| hsa-miR-3135b | 5 | 0.5230916 |
| hsa-miR-320b | 5 | 0.5160575 |
| eca-miR-30d | 5 | 0.5147498 |
| eca-miR-92b | 5 | 0.5140078 |
| bta-miR-107 | 5 | 0.5007385 |
| bta-miR-2478 | 5 | 0.4815487 |
| hsa-miR-30c-2-3p | 5 | 0.4740567 |
| eca-miR-186 | 5 | 0.473184 |
| eca-miR-181a | 5 | 0.4614267 |
| eca-miR-129a-5p | 5 | 0.4372207 |
| hsa-miR-30a-5p | 5 | 0.4230501 |
| hsa-miR-128-1-5p | 5 | 0.4200663 |
| hsa-miR-331-5p | 5 | 0.4114938 |
| bta-miR-1260b | 5 | 0.3570193 |
| bta-miR-151-3p | 5 | 0.3488351 |
| eca-miR-224 | 5 | 0.342172 |
| bta-miR-200b | 5 | 0.3391099 |
| eca-miR-10a | 5 | 0.3216599 |
| hsa-miR-4488 | 5 | 0.3185083 |
| eca-let-7d | 5 | 0.311156 |
| bta-let-7e | 5 | 0.310523 |
| bta-miR-29a | 5 | 0.2897291 |
| hsa-miR-1843 | 5 | 0.2815976 |
| hsa-miR-146b-5p | 5 | 0.2776504 |
| hsa-miR-574-3p | 5 | 0.2752821 |
| eca-miR-135b | 5 | 0.265792 |
| eca-miR-185 | 5 | 0.2631414 |
| eca-miR-598 | 5 | 0.2609939 |
| bta-miR-329b | 5 | 0.2539798 |
| bta-miR-11987 | 5 | 0.2533471 |
| eca-let-7f | 5 | 0.2531762 |
| hsa-miR-181c-3p | 5 | 0.2350728 |
| hsa-miR-7706 | 5 | 0.2340628 |
| eca-miR-200a | 6 | 0.8408441 |
| eca-miR-31 | 6 | 0.8355559 |
| bta-miR-139 | 6 | 0.8137145 |
| hsa-miR-16-2-3p | 6 | 0.8051487 |
| bta-miR-150 | 6 | 0.7537257 |
| eca-miR-24 | 6 | 0.6778058 |
| hsa-miR-203a-3p | 6 | 0.6574547 |
| bta-miR-146b | 6 | 0.6335661 |
| eca-miR-106b | 6 | 0.6239816 |
| eca-miR-155 | 6 | 0.5902864 |
| eca-miR-32 | 6 | 0.5482378 |
| eca-miR-142-3p | 6 | 0.5303207 |
| bta-miR-27a-3p | 6 | 0.4632714 |
| hsa-miR-135a-2-3p | 6 | 0.4603454 |
| hsa-miR-106b-3p | 6 | 0.4315356 |
| hsa-miR-339-3p | 6 | 0.4184814 |
| eca-miR-23a | 6 | 0.4176559 |
| eca-miR-20a | 6 | 0.4101832 |
| bta-miR-21-5p | 6 | 0.4081539 |
| hsa-miR-339-5p | 6 | 0.4019935 |
| bta-miR-140 | 6 | 0.369406 |
| eca-miR-205 | 6 | 0.3589372 |
| eca-miR-423-5p | 6 | 0.3555108 |
| eca-miR-21 | 6 | 0.355123 |
| bta-miR-17-5p | 6 | 0.3412015 |
| hsa-miR-335-3p | 6 | 0.3401004 |
| hsa-miR-675-3p | 6 | 0.3398162 |
| hsa-miR-7977 | 6 | 0.337607 |
| eca-miR-340-3p | 6 | 0.337242 |
| hsa-miR-455-5p | 6 | 0.3322791 |
| hsa-miR-3158-3p | 6 | 0.3283116 |
| bta-miR-451 | 6 | 0.3267909 |
| eca-miR-486-5p | 6 | 0.3262406 |
| eca-miR-92a | 6 | 0.3098255 |
| eca-miR-126-3p | 6 | 0.2967873 |
| eca-miR-146b-5p | 6 | 0.2774006 |
| eca-miR-26a | 6 | 0.2739876 |
| eca-miR-218 | 6 | 0.2625789 |
| hsa-miR-375-3p | 6 | 0.2565761 |
| bta-miR-26b | 6 | 0.2400987 |
| eca-miR-25 | 6 | 0.2302879 |
| eca-miR-142-5p | 6 | 0.2148911 |
| hsa-miR-21-3p | 6 | 0.2135385 |
| eca-miR-454 | 6 | 0.2096088 |
| eca-miR-193a-5p | 6 | 0.1953759 |
| eca-miR-15b | 6 | 0.187967 |
| eca-miR-374a | 6 | 0.1867033 |
| bta-miR-218 | 7 | 0.8026455 |
| eca-miR-187 | 7 | 0.7767203 |
| eca-miR-1 | 7 | 0.7760556 |
| hsa-miR-325 | 7 | 0.7020845 |
| eca-miR-330 | 7 | 0.6546666 |
| hsa-miR-9-3p | 7 | 0.6527925 |
| bta-miR-423-3p | 7 | 0.5952297 |
| hsa-miR-148a-5p | 7 | 0.5666093 |
| eca-miR-29c | 7 | 0.5503868 |
| hsa-miR-589-5p | 7 | 0.5416354 |
| hsa-miR-1271-5p | 7 | 0.5336122 |
| hsa-miR-615-3p | 7 | 0.5150091 |
| hsa-miR-374a-3p | 7 | 0.5048015 |
| eca-miR-335 | 7 | 0.4400164 |
| eca-miR-124 | 7 | 0.4324143 |
| eca-miR-374b | 7 | 0.4273627 |
| bta-miR-411a | 7 | 0.4253202 |
| bta-miR-219-3p | 7 | 0.4168937 |
| eca-miR-139-5p | 7 | 0.4030065 |
| bta-miR-196b | 7 | 0.3801705 |
| eca-miR-194 | 7 | 0.3765192 |
| hsa-miR-431-3p | 7 | 0.3747309 |
| bta-miR-30d | 7 | 0.3709614 |
| eca-miR-34c | 7 | 0.3667042 |
| hsa-miR-425-5p | 7 | 0.3654748 |
| eca-miR-214 | 7 | 0.3651635 |
| eca-miR-99b | 7 | 0.3484743 |
| bta-miR-421 | 7 | 0.3463337 |
| hsa-miR-503-5p | 7 | 0.3461627 |
| bta-miR-16b | 7 | 0.3456223 |
| hsa-miR-340-3p | 7 | 0.3420204 |
| bta-miR-181a | 7 | 0.3371302 |
| bta-miR-654 | 7 | 0.3328884 |
| bta-miR-129 | 7 | 0.3271595 |
| hsa-miR-181a-2-3p | 7 | 0.3170191 |
| bta-miR-22-5p | 7 | 0.314709 |
| eca-miR-872 | 7 | 0.3121434 |
| hsa-miR-382-3p | 7 | 0.2920216 |
| eca-miR-382 | 7 | 0.2900704 |
| hsa-miR-411-3p | 7 | 0.2888072 |
| eca-miR-182 | 7 | 0.2849069 |
| eca-miR-107b | 7 | 0.283192 |
| bta-miR-378c | 7 | 0.2810408 |
| eca-miR-379 | 7 | 0.271362 |
| eca-miR-30e | 7 | 0.263641 |
| eca-miR-134 | 7 | 0.2546219 |
| eca-miR-34a | 7 | 0.2534866 |
| eca-miR-660 | 7 | 0.2487166 |
| eca-miR-9a | 7 | 0.2480078 |
| eca-miR-409-5p | 7 | 0.2423341 |
| bta-miR-23b-3p | 7 | 0.234478 |
| hsa-miR-127-5p | 7 | 0.2277838 |
| eca-miR-411 | 7 | 0.2255086 |
| eca-miR-16 | 7 | 0.2190823 |
| hsa-miR-4516 | 7 | 0.2120071 |
| bta-miR-224 | 7 | 0.1990315 |
| eca-miR-141 | 8 | 0.7602035 |
| eca-miR-197 | 8 | 0.7570877 |
| eca-miR-708 | 8 | 0.7170864 |
| hsa-miR-3960 | 8 | 0.5996063 |
| eca-miR-149 | 8 | 0.5977978 |
| eca-miR-19b | 8 | 0.4978355 |
| eca-miR-1307 | 8 | 0.4107191 |
| eca-miR-128 | 8 | 0.4049803 |
| eca-miR-362-5p | 8 | 0.3771015 |
| bta-miR-2428 | 8 | 0.3641856 |
| eca-miR-450c | 8 | 0.3631474 |
| eca-miR-215 | 8 | 0.2627135 |
| bta-miR-7 | 8 | 0.2556731 |
| eca-miR-485-5p | 8 | 0.2335172 |
| eca-miR-95 | 8 | 0.2162284 |
| eca-miR-132 | 8 | 0.203144 |
| bta-miR-192 | 8 | 0.1983168 |
| bta-miR-215 | 8 | 0.1970722 |
| eca-miR-139-3p | 8 | 0.1936847 |
| bta-miR-202 | 8 | 0.1845993 |
| eca-miR-331 | 8 | 0.1834765 |
| hsa-miR-320c | 8 | 0.1805146 |
| hsa-miR-1224-5p | 8 | 0.1775608 |
| hsa-miR-3196 | 8 | 0.1733667 |
| hsa-miR-451a | 9 | 0.8056906 |
| eca-let-7a | 9 | 0.8050581 |
| eca-miR-30b | 9 | 0.7743082 |
| eca-miR-138 | 9 | 0.6798446 |
| eca-miR-96 | 9 | 0.6481514 |
| hsa-miR-221-5p | 9 | 0.6147375 |
| eca-miR-100 | 9 | 0.5698496 |
| eca-miR-409-3p | 9 | 0.566897 |
| eca-miR-125b-5p | 9 | 0.5527436 |
| hsa-miR-152-3p | 9 | 0.5435519 |
| eca-miR-199a-3p | 9 | 0.5251355 |
| hsa-miR-493-5p | 9 | 0.5012367 |
| hsa-miR-1180-3p | 9 | 0.5000022 |
| eca-miR-137 | 9 | 0.4677884 |
| bta-miR-126-3p | 9 | 0.4482854 |
| bta-miR-484 | 9 | 0.4271762 |
| hsa-miR-145-3p | 9 | 0.4123421 |
| eca-miR-542-3p | 9 | 0.3987363 |
| eca-miR-652 | 9 | 0.3912573 |
| bta-miR-125a | 9 | 0.3514166 |
| eca-miR-199b-5p | 9 | 0.3376608 |
| bta-miR-27a-5p | 9 | 0.3323102 |
| bta-miR-152 | 9 | 0.3306744 |
| bta-miR-574 | 9 | 0.3189707 |
| eca-miR-199a-5p | 9 | 0.3106804 |
| bta-miR-199c | 9 | 0.3070652 |
| hsa-miR-501-3p | 9 | 0.2987514 |
| bta-miR-375 | 9 | 0.2966062 |
| eca-miR-495 | 9 | 0.287145 |
| eca-miR-676 | 9 | 0.2839961 |
| eca-miR-361-5p | 9 | 0.2825216 |
| bta-miR-455-5p | 9 | 0.2822246 |
| eca-miR-369-3p | 9 | 0.2795705 |
| eca-miR-125a-5p | 9 | 0.2734168 |
| eca-miR-432 | 9 | 0.273271 |
| bta-miR-30e-5p | 9 | 0.2668922 |
| bta-miR-339a | 9 | 0.2634085 |
| bta-miR-22-3p | 9 | 0.2624844 |
| bta-miR-503-5p | 9 | 0.2574993 |
| eca-miR-532-3p | 9 | 0.2544189 |
| bta-miR-323 | 9 | 0.2399536 |
| eca-miR-450b-5p | 9 | 0.2366268 |
|  |  |  |
